# Dynamic Stability in Intermittent Seawater Electrolysis Via Frustrated Lewis Pair Engineering

**DOI:** 10.1002/advs.202518514

**Published:** 2025-11-25

**Authors:** Peilin Shen, Jiawei Zhu, Chen Deng, Shangqian Zhu, Xiaoman He, Wenguang Ouyang, Xin Tu, Huiyan Zhang, Richen Lin

**Affiliations:** ^1^ Key Laboratory of Energy Thermal Conversion and Control of the Ministry of Education School of Energy and Environment Southeast University Nanjing 211189 China; ^2^ School of Chemistry and Chemical Engineering Southeast University Nanjing 211189 China; ^3^ Department of Electrical Engineering and Electronics University of Liverpool Liverpool L69 3GJ UK

**Keywords:** frustrated lewis pairs, hydrogen evolution reaction, intermittent operation, seawater electrolysis

## Abstract

Alkaline seawater electrolysis powered by intermittent renewable energy offers a promising pathway for sustainable hydrogen production, yet faces critical challenges in proton supply dynamics and catalyst stability. The dual limitations are addressed through the design of a Cr‐NiCoP_v_@NF (P_v_: P vacancy, NF: nickel foam) electrocatalyst featuring frustrated Lewis pairs (FLPs). The metal‐phosphorus FLP architecture demonstrates ultralow overpotentials of 110 mV at the current density of 10 mA cm^−2^ and 333 mV at an industrial‐grade current density of 1 A cm^−2^ in a 1.0 m KOH + seawater electrolyte. Key innovation lies in the system's dynamic stability to intermittent operation, maintaining ≈100% activity after 520 h at 0.5 A cm^−2^ with 12 h start‐shutdown cycles. Combined experimental and theoretical analyzes reveal two crucial mechanisms: 1) FLPs synergistically facilitate H─OH bond dissociation (0.18 eV barrier reduction) and optimize hydrogen desorption energetics (0.13 eV barrier reduction), solving the proton supply limitation. 2) The selective adsorption behavior enables surface‐enriched OH^−^ groups to form a molecular‐level protective shield that repels chlorides through electrostatic effects, effectively mitigating catalyst corrosion. This work establishes a new paradigm for non‐precious metal catalyst design via targeted electronic structure engineering, while providing fundamental insights into the interfacial microenvironment under intermittent operations.

## Graphical abstract

The Frustrated Lewis Pair concept guides the design strategy of Cr‐NiCoP_v_@NF to address dual limitations in intermittent alkaline seawater electrolysis.

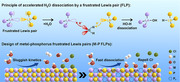

## 1 Introduction

Renewable energy‐driven green hydrogen production through large‐scale seawater electrolysis has emerged as a global research priority for energy transition.^[^ ^1^, ^2^, ^3^, ^4^, ^5^ ^]^ This approach not only circumvents freshwater scarcity concerns but also enables direct integration with offshore renewable energy infrastructure.^[^ ^6^ ^]^ However, the practical implementation of seawater electrolysis faces formidable technical barriers stemming from the complex ionic composition of seawater (e.g., Cl^−^, Br^−^, Ca^2+^, Mg^2+^), which induces severe catalyst deactivation through multiple pathways, including competitive chloride oxidation, cathodic deposition, and corrosive attack on electrode materials.^[^ ^7^ ^]^ Alkaline media (pH > 9.5) can establish an oxygen evolution reaction (OER) selectivity window of ≈0.48 V to suppress chlorine oxidation reactions,^[^ ^8^ ^]^ while pre‐precipitation of insoluble Ca(OH)_2_ and Mg(OH)_2_ can effectively mitigate cathodic active site blockage,^[^ ^9^, ^10^ ^]^ positioning alkaline seawater electrolysis as a promising research direction. Despite this theoretical framework, industrial adoption confronts two fundamental challenges: 1) The hydrogen evolution reaction (HER) kinetics in alkaline environments suffer from intrinsic sluggishness due to the rate‐limiting water dissociation step (Volmer step), with reaction rates typically two orders of magnitude lower than in acidic systems.^[^ ^11^, ^12^ ^]^ This kinetic bottleneck arises from the limited proton availability in alkaline conditions, requiring catalysts to first split water molecules before hydrogen adsorption can occur. 2) The intermittent nature of renewable energy sources (e.g., solar and wind) necessitates electrolyzers that can withstand frequent start‐shutdown cycles. During the shutdown period, cathode materials are exposed to oxidation potentials that trigger chloride adsorption and subsequent corrosion.^[^ ^9^, ^13^ ^]^

Recent advances in nanomaterial engineering have accelerated the development of cost‐effective transition metal (TM)‐based cathode catalysts for alkaline seawater hydrogen evolution reactions. A diverse array of TM compounds, including sulfides,^[^ ^14^, ^15^, ^16^ ^]^ phosphides,^[^ ^9^, ^17^, ^18^, ^19^ ^]^ oxides,^[^ ^20^, ^21^ ^]^ nitrides,^[^ ^3^, ^22^ ^]^ and tellurides,^[^ ^23^ ^]^ have demonstrated promising catalytic performance through various design strategies, such as heterostructures,^[^ ^9^, ^14^, ^15^, ^17^, ^22^, ^23^, ^24^ ^]^ elemental doping,^[^ ^18^, ^20^, ^21^ ^]^ ionic intercalation,^[^ ^14^ ^]^ built‐in electric fields,^[^ ^17^ ^]^ and phase transitions.^[^ ^25^, ^26^, ^27^ ^]^ These approaches have primarily focused on steady‐state performance optimization, but their dynamic stability under intermittent operational conditions remains insufficiently explored. The design of cathode catalysts capable of simultaneously reducing the HER activation energy while resisting chloride‐induced corrosion during shutdown periods remains a significant challenge.

**Figure 1:**
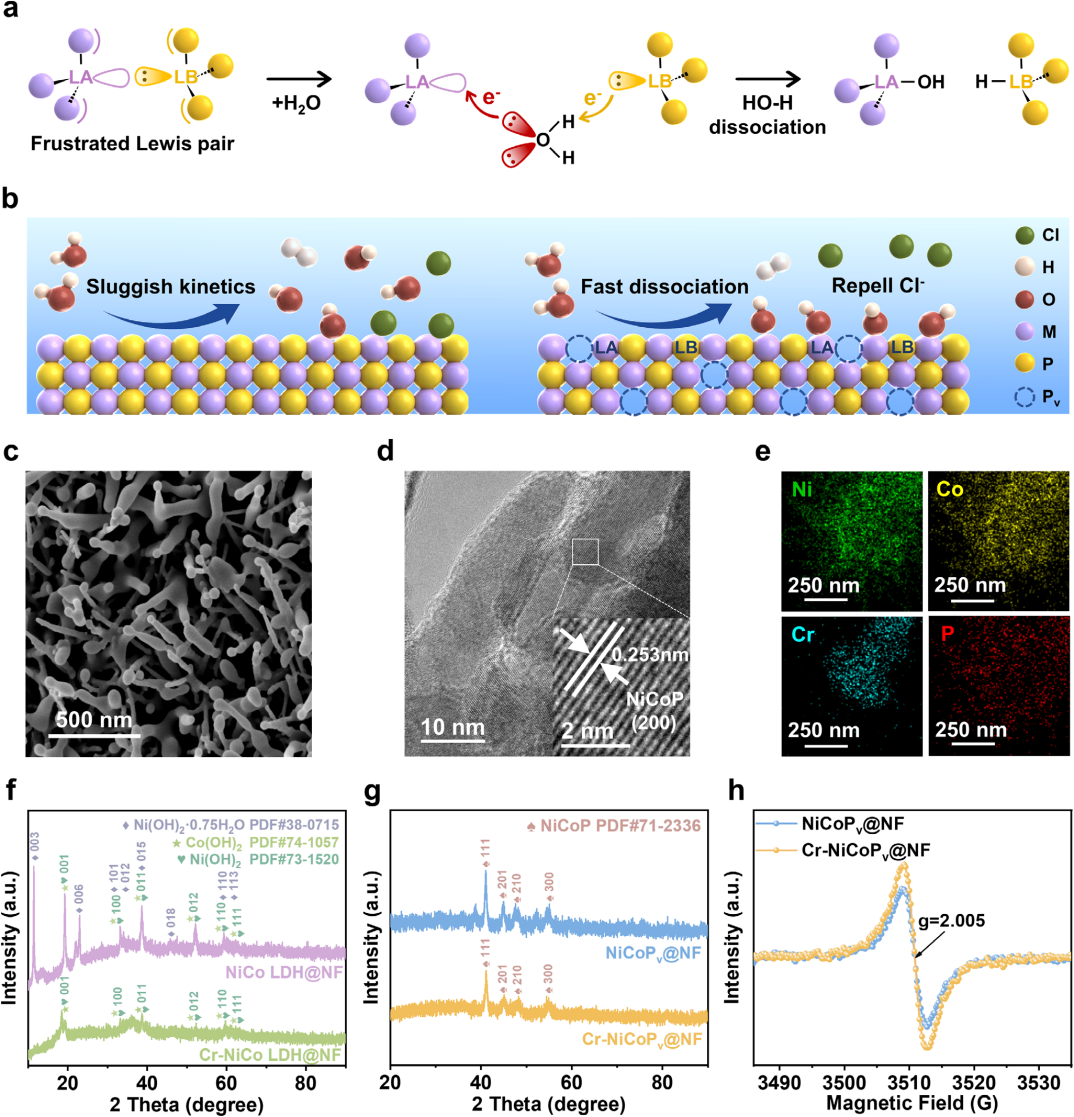
Design strategy and structural characterization of the Cr‐NiCoP_v_@NF. a) Possible electron transfer mechanism of dissociating H_2_O by FLP. b) Schematic diagram of the designed M‐P FLPs electrocatalyst. M represents the metal‐atom Cr, Ni, or Co. c) SEM image of Cr‐NiCoP_v_@NF. d) HRTEM image of Cr‐NiCoP_v_@NF. The insert shows the magnified HRTEM images corresponding to the regions depicted in Figure 1d. e) Element dispersive spectroscopy of Cr‐NiCoP_v_@NF. f) XRD patterns of NiCo LDH@NF and Cr‐NiCo LDH@NF. g) XRD patterns of NiCoP_v_@NF and Cr‐NiCoP_v_@NF. h) EPR spectra of NiCoP_v_@NF and Cr‐NiCoP_v_@NF. a.u., arbitrary units.

The emerging concept of frustrated Lewis pairs (FLP) has recently garnered remarkable attention,^[^ ^12^, ^28^, ^29^, ^30^, ^31^, ^32^, ^33^, ^34^, ^35^, ^36^, ^37^ ^]^ providing a novel opportunity to address the above‐mentioned challenges. In chemical terms, a Lewis acid (LA) is defined as an electron‐pair acceptor characterized by vacant orbitals or electron‐deficient centers, while a Lewis base (LB) represents an electron‐pair donor featuring lone electron pairs or unshared electron densities. Classical Lewis acid‐base interactions result in electron pair donation from the LB to the LA, forming a stable covalent bond (Figure S1a, Supporting Information). However, in FLP systems, steric constraints prevent this covalent bond formation (Figure S1b, Supporting Information). Due to the steric hindrance effect, the LA and LB cannot form stable adducts, thus retaining their respective properties and enabling the synergistic activation of small molecules. Building upon this fundamental principle, we hypothesize an innovative “metal‐phosphorus frustrated Lewis pairs (M‐P FLPs)” design paradigm with abundant surface atomic defects. In this architecture, the metal (M) atom adjacent to a P vacancy (P_v_) serves as the LA center, while the non‐bonded P atom surrounding the P vacancy functions as the LB center. This configuration exhibits unique potential for water molecule activation: the electron‐rich oxygen atoms (with lone electron pairs) may react with LA sites and form chemical bonds, while the electron‐deficient hydrogen atoms may react with LB sites and form chemical bonds, thereby synergistically promoting water dissociation (**Figure** **1**a). This cooperative action between the LA and LB sites facilitates the heterolytic cleavage of the H─OH bond, thereby dramatically lowering the kinetic barrier of the water dissociation step. Furthermore, based on the hard‐soft acid‐base (HSAB) theory, harder Lewis acids preferentially bind to harder bases. Since OH^−^ is a harder Lewis base than Cl^−^,^[^ ^37^, ^38^, ^39^ ^]^ when the M site as the Lewis base site is hard enough, its affinity for OH^−^ will be stronger than that for Cl^−^, while LB sites have approximately no affinity for Cl^−^since chloride ions also belong to Lewis bases. This hypothetically reduces the adsorption of Cl^−^ on the electrode surface during the shutdown phase, thus breaking through the bottleneck of the instability of conventional catalysts under intermittent operation conditions. This innovative conceptual design may simultaneously address both the kinetic limitations of alkaline HER and the stability challenges posed by intermittent operation.

In this work, we first designed the NiCoP_v_@NF (P_v_: P vacancy, NF: nickel foam) electrode with FLP active sites by using Ni/Co as the frustrated LA sites and P as the frustrated LB sites. Guided by the HSAB theory, we hypothesized that further introducing Cr^3+^ (the hardest Lewis acid among transition metals) would strengthen OH^−^ (harder Lewis base than Cl^−^) binding with M sites (LA sites),^[^ ^7^ ^]^ thereby enhancing water dissociation and the resistance to chloride ions. To validate this, we engineered the Cr‐NiCoP_v_@NF electrode (Figure 1b). The Cr‐NiCoP_v_@NF demonstrates exceptional performance in alkaline seawater electrolysis. The HER overpotentials in 1.0 m KOH alkaline electrolyte and 1.0 m KOH + seawater mixed system were only 90 and 110 mV at a current density of 10 mA cm^−2^, respectively. The overpotentials remained at excellent levels of 328 and 333 mV when the current density was increased to the industrial grade of 1 A cm^−2^. The anion‐exchange membrane (AEM) electrolyzer maintains continuous performance for over 500 h at 0.2 A cm^−2^, and enduring rigorous intermittent operation (12‐h start‐shutdown cycles) for over 520 h at 0.5 A cm^−2^ with negligible performance degradation. This work demonstrates a FLP‐based design paradigm for the development of high‐activity, corrosion‐resistant electrocatalysts, advancing industrial seawater electrolysis for intermittent operations.

## 2 Results

### 2.1 FLP Electrocatalyst Synthesis and Characterization

To implement the FLP‐based design, Cr‐NiCoP_v_@NF electrocatalysts were synthesized via an optimized two‐step protocol that precisely controls phosphorus vacancy concentration while ensuring structural stability. In the first stage, a chromium‐doped nickel‐cobalt layered double hydroxide (Cr‐NiCo LDH) precursor was grown on pretreated nickel foam substrates via a precisely controlled hydrothermal reaction at 120 °C for 6 h. This process yielded a Cr‐NiCo LDH@NF intermediate with an interlocking nanoneedle architecture. The second stage involved a gas‐phase phosphorization process conducted at 350 °C for 2 h under a controlled Ar atmosphere using sodium hypophosphite (NaH_2_PO_2_) as the phosphorus source, the precursor was converted into the target catalyst Cr‐NiCoP_v_@NF. In our pre‐experiment, we optimized the incorporation amount of Cr. The Cr/(Ni+Co) molar ratio of 0.3/2 (15%) was identified as the optimal dosage for this specific synthesis system and was consequently selected for the further study presented. This optimal ratio likely represents the best balance between creating a sufficient number of active FLP sites and maintaining the structural and electronic integrity of the NiCoP host lattice. Scanning electron microscopy (SEM) analysis revealed that the phosphorized Cr‐NiCoP_v_@NF presented a nanorod morphology (Figure 1c), and its surface exhibited pronounced tip‐passivation characteristics compared to the precursor. Comparative SEM examination of the catalysts indicated that trace Cr doping had no significant influence on electrode morphology (Figure S2, Supporting Information). The 3D nanorod architecture substantially increased the electrode's specific surface area, providing abundant active sites for the HER and potentially efficient gas bubble release channels. The low‐magnification transmission electron microscopy (TEM) observations of Cr‐NiCoP_v_@NF further confirmed the nanorod structural consistency with SEM results (Figure S3, Supporting Information). High‐resolution TEM (HRTEM) imaging of Cr‐NiCoP_v_@NF revealed distinct lattice fringes with a spacing of 0.253 nm, corresponding to the (200) crystallographic plane of NiCoP (PDF#71‐2336) (Figure 1d), confirming the formation of a NiCoP dominant crystalline phase after phosphorization, and the P vacancy creation enabled the formation of catalytically active FLP sites without compromising NiCoP crystalline integrity. Additionally, energy dispersive spectroscopy (EDS) elemental mapping demonstrated a homogeneous spatial distribution of Ni, Co, P, and Cr within the material (Figures 1e; S4, Supporting Information), confirming successfull incorporation of Cr into the host lattice without surface segregation.

The crystal structure and defect properties of the materials were systematically characterized using X‐ray diffraction (XRD) and electron paramagnetic resonance (EPR). The XRD patterns revealed that the NiCo LDH@NF precursor primarily consisted of Ni(OH)_2_ (PDF#73‐1520), Ni(OH)_2_·0.75H_2_O (PDF#38‐0715), and Co(OH)_2_ (PDF#74‐1057) (Figure 1f). In contrast, only the characteristic peaks of Ni(OH)_2_ and Co(OH)_2_ remained in Cr‐NiCo LDH@NF, suggesting that Cr doping may suppress the formation of the hydrated phase. Following phosphorization, both NiCoP_v_@NF and Cr‐NiCoP_v_@NF exhibited distinct NiCoP (PDF#71‐2336) diffraction peaks (Figure 1g), confirming the successful conversion of the precursor into the phosphide phase. Notably, Cr doping did not cause significant shifts in the lattice parameter. Combined with the previously mentioned HRTEM analysis, it is indicated that the incorporation of trace amounts of Cr would not disrupt the lattice structure of NiCoP. The EPR spectra displayed nearly centrosymmetric signal peaks centered at g = 2.005 for both NiCoP_v_@NF and Cr‐NiCoP_v_@NF (Figure 1h), confirming the presence of the same type of unpaired electrons, which could reflect the existence of P vacancies.^[^ ^40^ ^]^ Quantitative analysis revealed that the EPR peak intensities of Cr‐NiCoP_v_@NF are significantly higher than NiCoP_v_@NF, suggesting an increased number of P vacancy sites in Cr‐NiCoP_v_@NF.^[^ ^41^ ^]^ This result clearly demonstrates that Cr doping effectively promotes P vacancy formation. The Lewis acid (Cr/Ni/Co cations) and Lewis base (‐OH groups) sites in the unphosphorized precursor can exist in principle. To further investigate the construction of FLPs on the catalyst surface after phosphorization, CO_2_ temperature programmed desorption (TPD) and NH_3_ TPD experiments were conducted.^[^ ^12^, ^34^ ^]^ As shown in Figure S5 (Supporting Information), the phosphorized samples exhibited CO_2_ desorption peaks at ≈450 and 710 °C. Compared with the unphosphorized precursor, the CO_2_ TPD spectra showed the low‐temperature peak shifted to higher temperatures, and a new high‐temperature peak emerged, suggesting enhanced Lewis basicity on the material surface after phosphorization. Similarly, the NH_3_ TPD spectra of the phosphorized samples showed a marked increase in the intensity of Lewis acidic sites. EPR spectroscopy confirms the presence of P vacancies, which provides the structural prerequisite for FLPs. Importantly, TPD results reveal a simultaneous enhancement in both Lewis acidity and basicity after phosphidation, which contradicts the expected activity quenching due to classical Lewis acid‐base adduct formation but is consistent with the characteristic independent and synergistic reactivity of FLPs maintained by steric hindrance. Collectively, these EPR and TPD results confirmed the successful formation of FLPs on the surfaces of the NiCoP_v_@NF and Cr‐NiCoP_v_@NF catalytic electrodes.

**Figure 2:**
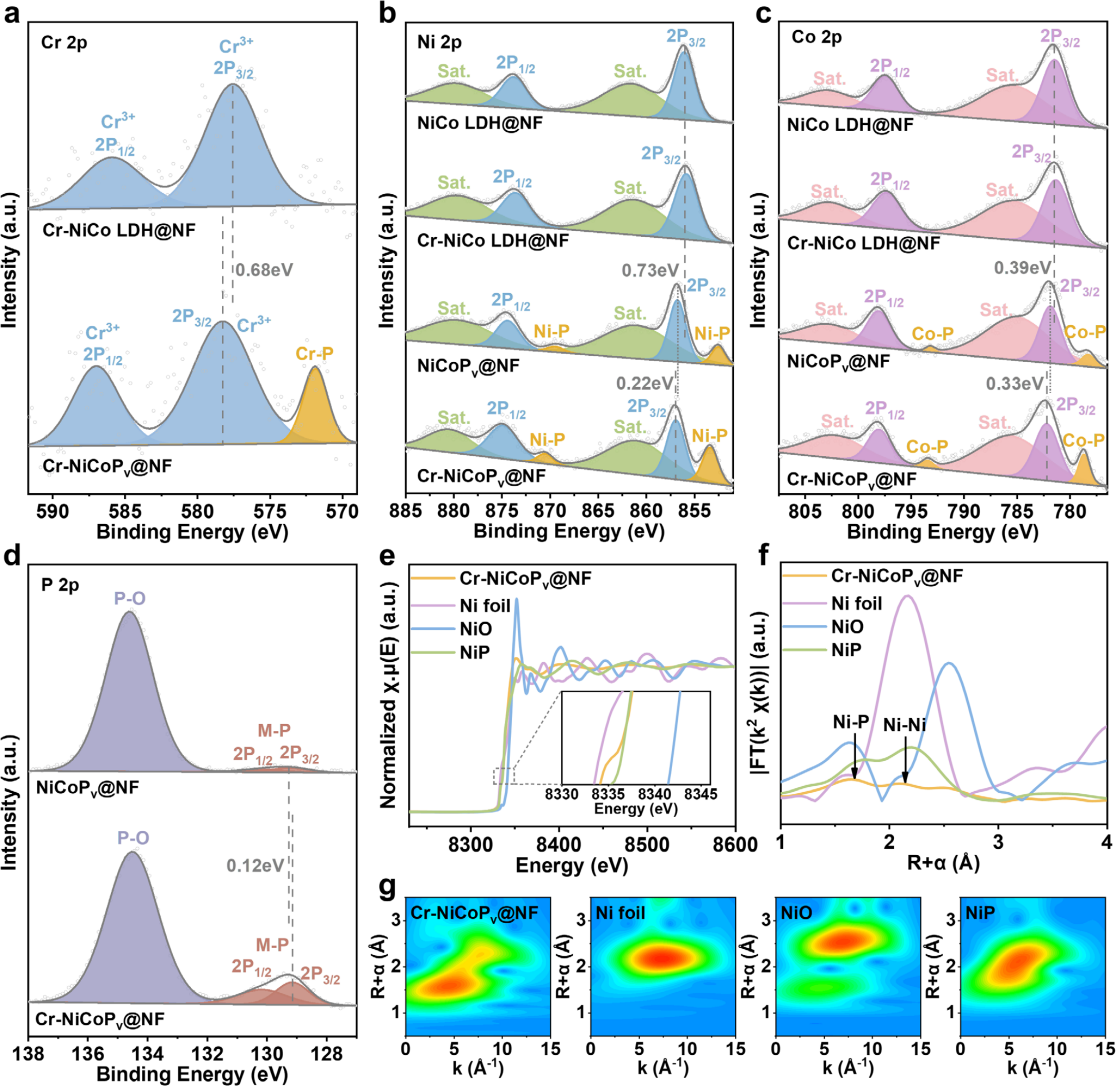
Electronic and fine‐structure characterizations of the Cr‐NiCoP_v_@NF and control samples. High‐resolution a) Cr 2p, b) Ni 2p, c) Co 2p, and d) P 2p XPS spectra. e) Ni K‐edge XANES spectra. f) FT‐EXAFS spectra. g) Wavelet transform‐EXAFS spectra.

The electronic structures of Cr‐NiCoP_v_@NF and its control samples were further investigated using high‐resolution X‐ray photoelectron spectroscopy (XPS). The XPS survey further confirmed the presence of Ni, Co, and other major elements in the catalysts. A comparative analysis of the high‐resolution spectra before and after phosphorization revealed significant positive binding energy shifts in the Cr 2p, Ni 2p, and Co 2p orbitals following phosphorization, indicating that phosphorization effectively restructures the electronic environment of the metal centers and induces a more electron‐deficient state of the metal center. The new characteristic peaks Cr‐P (≈571.8 eV), Ni‐P (≈853.4 eV), and Co‐P (≈778.6 eV) emerged after phosphorization,^[^ ^40^, ^42^, ^43^, ^44^, ^45^ ^]^ confirming the successful conversion of the precursor into the phosphide phase. The Cr 2p high‐resolution spectrum revealed that the introduced Cr element is mainly in the positive trivalent oxidation state,^[^ ^46^ ^]^ belonging to the hard Lewis acid site (**Figure** **2**a). Furthermore, compared with NiCoP_v_@NF, Cr doping induced additional positive shifts in the Ni 2p_3/2_ and Co 2p_3/2_ binding energies by ≈0.22 and 0.33 eV, respectively (Figure 2b,c). The increase in the binding energy of Ni 2p and Co 2p confirmed that Cr^3+^ incorporation synergistically enhances the Lewis acidity of Ni and Co sites. A P‐O peak about 134.4 eV was observed in the P 2p high‐resolution spectrum (Figure 2d), likely due to slight oxidation on the surface upon air exposure.^[^ ^42^ ^]^ The characteristic peaks that occurred at 130.2 eV (2p_1/2_) and 129.1 eV (2p_3/2_) correspond to the formation of M─P bonds. Intriguingly, Cr doping caused a negative shift of ≈0.12 eV of the corresponding binding energy of P 2p_3/2_ characteristic peaks, suggesting enhanced Lewis basicity at the P sites due to electron transfer from metal centers. XPS reveals a “bidirectional polarization” in electronic structure. Cr doping induces a positive binding energy shift at the metal sites (enhanced Lewis acidity) and a negative shift at the P sites (enhanced Lewis basicity). This electronic restructuring represents an ideal configuration for constructing highly effective FLPs, indicating that the acid and base sites are co‐regulated within a cooperative system, rather than varying independently. Combined with the EPR and TPD results, these findings confirmed that Cr doping promotes the formation of higher‐density and more active FLP sites on the catalyst surface. Thus, Cr‐NiCoP_v_@NF can be regarded as an enhanced variant of the NiCoP_v_@NF frustrated Lewis pairs system.

To further elucidate the coordination environment of Cr‐NiCoP_v_@NF, we performed X‐ray absorption near‐edge structure (XANES) and extended X‐ray absorption fine structure (EXAFS) analyses. The Ni K‐edge XANES spectra revealed that the pre‐edge peak intensity and absorption edge position suggest an average oxidation state of nickel in Cr‐NiCoP_v_@NF intermediate between metallic Ni^0^ and Ni^2+^ (Figure 2e). Fourier‐transform (FT) k^2^‐weighted EXAFS spectra exhibited characteristic peaks at ≈1.7 and 2.1 Å, corresponding to Ni─P coordination bonds and Ni─Ni metallic bonds, respectively (Figure 2f). This coordination environment was further confirmed by wavelet transform analysis (Figure 2g), which resolved these scattering paths with enhanced resolution in both R‐space and k‐space.

### 2.2 Electrocatalytic HER Performance

**Figure 3:**
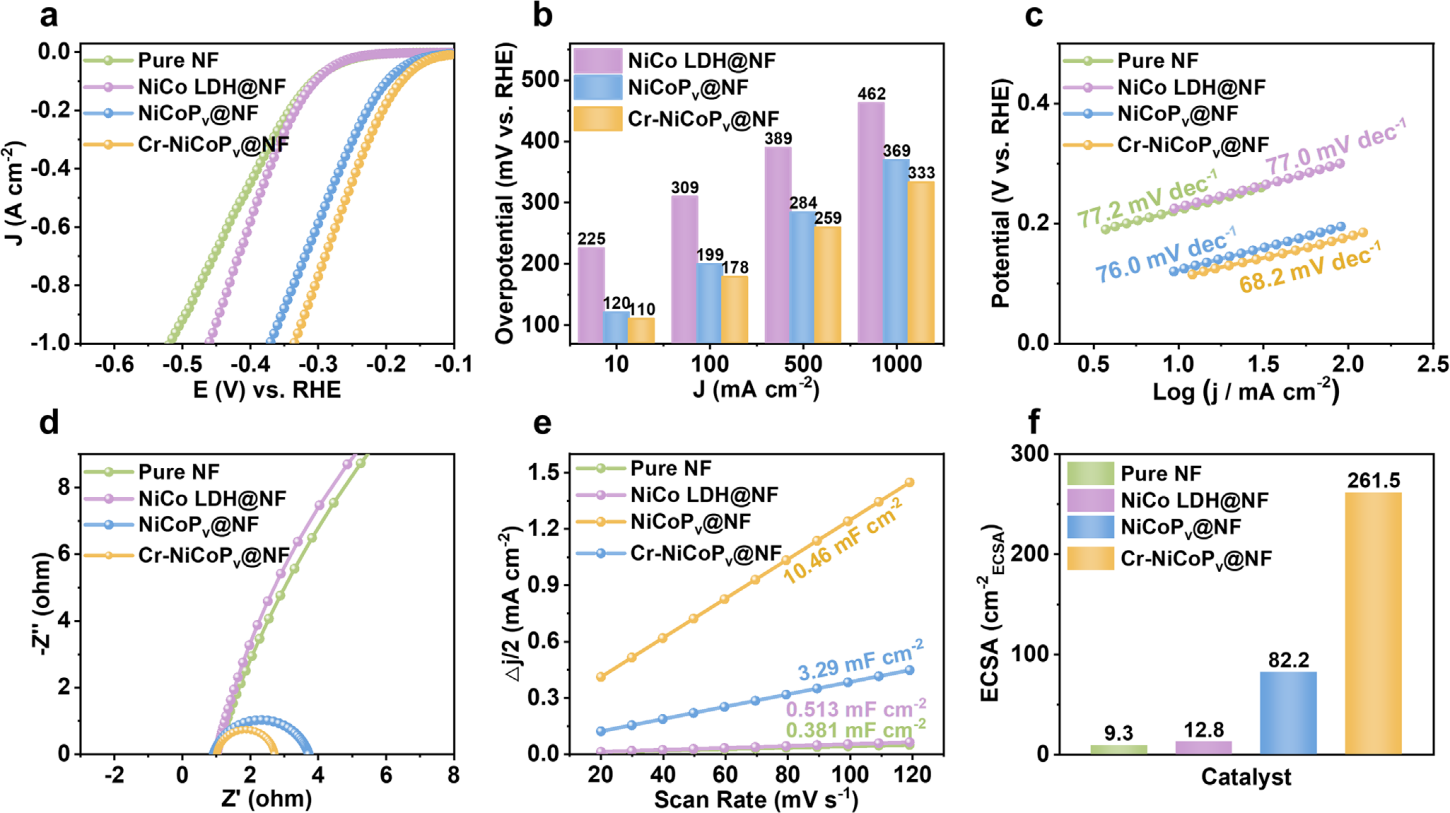
Electrochemical performance of Cr‐NiCoP_v_@NF and control samples in 1.0 m KOH + seawater electrolyte. a) Representative HER polarization curves. b) The overpotential at a current density of 0.01, 0.1, 0.5, and 1 A cm^−2^. c) Tafel slopes. d) Nyquist plots. e) Double‐layer capacitances. f) Electrochemically active surface area.

The HER performance of the synthesized catalysts was systematically evaluated in 1.0 m KOH + seawater electrolyte using a standard three‐electrode system. Linear sweep voltammetry (LSV) polarization curves revealed that both phosphorized NiCoP_v_@NF and Cr‐NiCoP_v_@NF electrodes exhibited significantly enhanced HER activity, with the Cr‐doped sample demonstrating the most outstanding catalytic performance (**Figure** **3**a). Notably, Cr‐NiCoP_v_@NF required overpotentials of merely 110, 178, 259, and 333 mV to achieve current densities of 10, 100, 500, and 1000 mA cm^−2^, respectively (Figure 3b). These performance metrics significantly surpass those of comparable non‐precious metal catalysts reported in the literature (Table S1, Supporting Information), highlighting the exceptional catalytic efficiency of our material in seawater electrolysis applications. This performance enhancement can be attributed to the successful formation of M‐P FLPs through phosphorization treatment, with trace Cr doping further promoting FLP sites formation, consistent with the earlier EPR, TPD, and XPS characterization results. To elucidate the reaction kinetics, Tafel slopes were derived from the polarization curves (Figure 3c). Cr‐NiCoP_v_@NF exhibited the smallest Tafel slope (68.2 mV dec^−1^), indicating the most favorable HER kinetics. Electrochemical impedance spectroscopy (EIS) measurements provided further evidence for the superior charge transfer characteristics of Cr‐NiCoP_v_@NF (Figure 3d). The Nyquist plots, analyzed using equivalent circuit fitting, revealed that Cr‐NiCoP_v_@NF displayed the smallest semicircular arc among all samples, confirming its minimal charge transfer resistance. This dramatic reduction in charge transfer resistance directly correlates with the improved HER activity and confirms the enhanced electronic conductivity facilitated by FLPs formation. Furthermore, we determined the electrochemical double‐layer capacitance (C_dl_) from cyclic voltammetry (CV) scans in the non‐Faradaic region (Figures 3e; S6, Supporting Information). Cr‐NiCoP_v_@NF demonstrated a C_dl_ of 10.46 mF cm^−2^, significantly higher than control samples, reflecting its superior charge storage capacity. Calculation of electrochemically active surface area (ECSA) showed that Cr‐NiCoP_v_@NF possessed an ECSA of 261.5 cm^−2^_ECSA_, approximately threefold greater than NiCoP_v_@NF and over twenty times higher than other controls (Figure 3f). These results collectively confirm that Cr‐NiCoP_v_@NF features more abundant FLP sites and provides significantly greater active surface area for HER.

The HER performance of the catalysts was also systematically evaluated in 1.0 m KOH electrolyte using a standard three‐electrode system (Figures S7 and S8, Supporting Information). The results demonstrated excellent consistency with those obtained in 1.0 m KOH + seawater electrolyte, with Cr‐NiCoP_v_@NF exhibiting the highest catalytic activity, followed by NiCoP_v_@NF. The exceptional HER performance of Cr‐NiCoP_v_@NF provides compelling experimental validation for our proposed FLP mechanism and can be rationalized through the synergistic interplay of its distinctive structural and electronic characteristics. The intentionally engineered phosphorus vacancies create coordinatively unsaturated metal sites that function as effective Lewis acid centers, while the adjacent non‐bonded P atoms serve as complementary Lewis base sites, thereby forming the hypothesized M‐P FLPs. More importantly, the incorporation of Cr dopants induces significant electronic structure modulation, as evidenced by our EPR, TPD, and XPS analyses, which not only increases the density of FLP sites but also optimizes their catalytic functionality. The collective experimental evidence unambiguously demonstrates that FLP sites work in synergy to enhance HER catalytic performance, accelerate reaction kinetics, increase C_dl_ and ECSA, and reduce overpotential and charge transfer resistance.

### 2.3 FLP‐Mediated Electrocatalysis Mechanism

**Figure 4:**
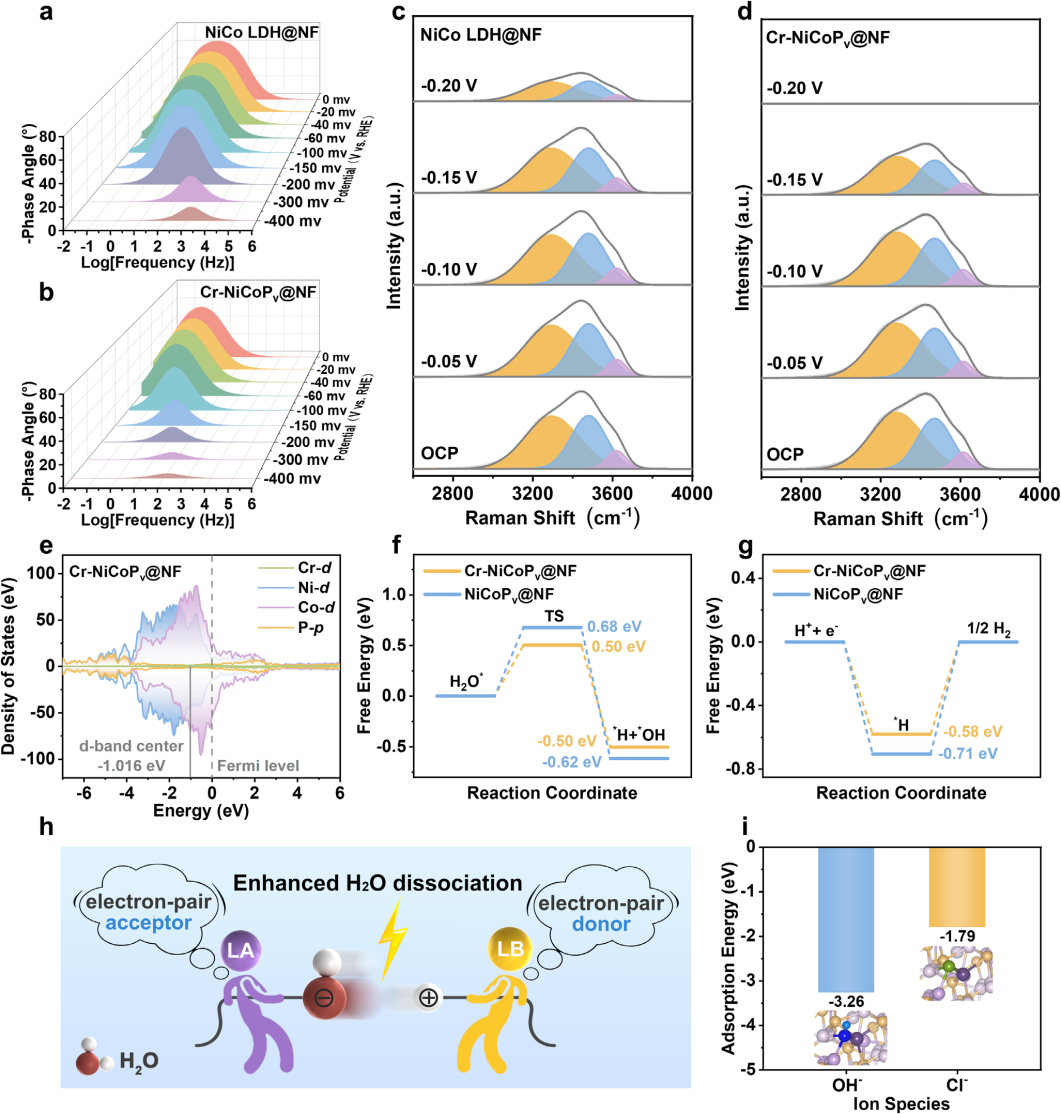
Investigation of the electrocatalytic mechanism of FLP. In situ Bode phase plots of a) NiCo LDH@NF and b) Cr‐NiCoP_v_@NF at different applied overpotentials in 1.0 m KOH + seawater electrolyte. In situ Raman spectra of interfacial water of c) NiCo LDH@NF and d) Cr‐NiCoP_v_@NF at different applied overpotentials in 1.0 m KOH + seawater electrolyte. OCP: open circuit potential. e) Density of states of Cr‐NiCoP_v_@NF. f) Gibbs free energy diagram of the water dissociation process. TS: transition state. g) Gibbs free energy diagram of the ^*^H desorption process. h) Catalytic mechanism diagram of Cr‐NiCoP_v_@NF during the water dissociation process. i) Comparison of Cl^−^ and OH^−^ adsorption energy on Cr‐NiCoP_v_@NF surfaces. The insets in Figure 4i are structure models of Cr‐NiCoP_v_@NF.

To elucidate the microscopic mechanism underlying the exceptional HER performance of Cr‐NiCoP_v_@NF, we conducted systematic in situ electrochemical impedance spectroscopy (EIS) measurements spanning a wide overpotential range (from 0 to −400 mV). The acquired spectra were rigorously analyzed using a physically meaningful equivalent circuit model that accounts for three critical electrochemical parameters: 1) solution resistance (R_s_) representing bulk electrolyte conductivity; 2) charge transfer resistance (R_ct_) characterizing the interfacial reaction kinetics; and 3) a constant‐phase element describing the non‐ideal capacitive behavior of the electrode‐electrolyte interface (Figure S9, Supporting Information). The in situ Nyquist plots demonstrate that Cr‐NiCoP_v_@NF exhibits significantly smaller R_ct_ values than NiCo LDH@NF at identical potentials (Figures S10 and S11, Supporting Information), directly confirming that the presence of FLPs markedly enhances interfacial charge transfer kinetics. Further insight into the catalytic process was obtained through in situ Bode plot analysis (**Figures** **4**a,b; S12 and S13, Supporting Information). The data reveal that Cr‐NiCoP_v_@NF displays the most rapid decay in phase angle with increasing overpotential (from 0 to −400 mV), reaching its minimum value at −400 mV. These trends are consistent precisely with the observed HER performance metrics, demonstrating that the FLP sites create highly efficient electronic pathways.^[^ ^47^ ^]^

In this study, in situ Raman spectroscopy was employed to monitor real‐time structural evolution of interfacial water during the HER at Cr‐NiCoP_v_@NF and NiCo LDH@NF electrodes (Figure 4c,d). The O‐H stretching vibration region (3000–3700 cm^−1^) was deconvoluted through Gaussian peak fitting into three distinct components corresponding to different water coordination states: tetrahedrally coordinated hydrogen‐bonded water (4‐HB‐H_2_O) representing strongly networked water molecules, doubly coordinated hydrogen‐bonded water (2‐HB‐H_2_O) indicating moderately bonded interfacial water, and free water molecules (Free‐H_2_O).^[^ ^48^, ^49^, ^50^, ^51^, ^52^, ^53^ ^]^ Notably, both catalysts exhibited significant attenuation of O‐H stretching intensities with increasingly negative applied potentials, attributed to accelerated consumption of interfacial water molecules participating in the HER process.^[^ ^51^ ^]^ Detailed analysis revealed that Cr‐NiCoP_v_@NF demonstrated more pronounced O‐H signal decay at potentials below −0.1 V, indicating faster water consumption and enhanced surface reactivity compared to NiCo LDH@NF, which maintained relatively slower O‐H signal decay kinetics. This distinct behavior originates from the synergistic effect of M‐P FLPs on the Cr‐NiCoP_v_@NF surface. While the Volmer step (water dissociation) is typically considered the rate‐determining step (RDS) in conventional alkaline electrolysis systems,^[^ ^11^, ^54^ ^]^ our findings suggest that the FLP engineering significantly accelerated the Volmer step.^[^ ^12^, ^55^ ^]^

To elucidate the mechanistic role of FLPs in the HER process, we performed density functional theory (DFT) calculations on the synthesized catalysts (Figures S14–S18, Supporting Information). Electronic structure analysis reveals that Cr‐NiCoP_v_@NF exhibits a d‐band center (−1.016 eV) closest to the Fermi level compared to NiCoP_v_@NF (−1.028 eV) and NiCo LDH@NF (−1.491 eV) (Figures 4e; S19, Supporting Information). This progressive shift in d‐band center position follows the well‐established d‐band theory of chemisorption, where the closer proximity to the Fermi level strengthens the interaction between the catalyst surface and reaction intermediates. This upward shift of the d‐band center significantly enhances the catalyst surface's interaction with reactants, consistent with our experimental observations of enhanced HER activity. Further examination of the density of states (DOS) provided additional insights into the superior catalytic properties of Cr‐NiCoP_v_@NF. DOS analysis further demonstrated that Cr‐NiCoP_v_@NF possesses the highest density of states near the Fermi level, indicating enhanced metallic character and superior electrical conductivity. This characteristic is particularly important for maintaining high catalytic activity under industrial‐scale current densities, as it ensures efficient electron transfer throughout the reaction process. Orbital‐projected charge density analysis showed strong hybridization between Ni/Co d‐orbitals and P p‐orbitals in phosphorization‐treated catalysts, while the introduction of Cr d‐orbitals in Cr‐NiCoP_v_@NF further optimizes electron delocalization and provides efficient charge transfer pathways. These electronic structure modifications created highly efficient charge transfer pathways that explain the experimentally observed low charge transfer resistance and the Cr‐NiCoP_v_@NF catalyst's ability to sustain high current densities at remarkably low overpotentials. The energy barrier analysis provided crucial mechanistic insights into how the FLP architecture enhances each step of the HER process. NiCoP_v_@NF required overcoming a relatively high energy barrier of 0.68 eV for water dissociation (Figure 4f), whereas the Cr‐doped M‐P FLP active sites in Cr‐NiCoP_v_@NF significantly reduced this barrier to 0.50 eV through enhanced synergistic effects. Notably, as an optimized version of the NiCoP_v_@NF FLP system, Cr‐NiCoP_v_@NF also lowers the hydrogen desorption energy barrier from 0.71 to 0.58 eV (Figure 4g).

The reduction of the water dissociation barrier after the addition of Cr element can be attributed to the FLP's unique synergistic mechanism, as illustrated in Figure 4h: the enhanced LA and LB sites form an electron‐pair acceptor‐donor system that significantly weakens water HO─H bonds through an electron “tug‐of‐war” effect, thereby dramatically enhancing hydrolysis kinetics. The reduced hydrogen desorption energy barrier stems from the favorable electronic structure of Cr‐NiCoP_v_@NF. Specifically, Cr doping enhances the Lewis basicity and electron density of P sites, promoting electron donation to weaken the H‐adsorbate bond. Furthermore, the high density of states near the Fermi level in Cr‐NiCoP_v_@NF ensures efficient electron transfer throughout this process. The theoretical framework developed in this study goes beyond explaining the exceptional performance of Cr‐NiCoP_v_@NF and establishes general design principles for FLP‐based electrocatalysts. The key features, optimal d‐band center positioning, efficient charge delocalization pathways, and spatially separated but cooperative active sites, can be adapted to other electrocatalytic systems facing similar challenges.

DFT calculation of adsorption energy revealed important interfacial properties of Cr‐NiCoP_v_@NF. Figure 4i demonstrated a striking difference in the interaction strengths between the catalyst surface and various anions, the adsorption energy of OH^−^ (−3.26 eV) was significantly lower than that of Cl^−^ (−1.79 eV) on the surface of Cr‐NiCoP_v_@NF. The introduction of Cr enhances the adsorption of OH^−^ on the surface while simultaneously suppressing the adsorption of Cl^−^ (Figure S20, Supporting Information). The occurrence of this selective adsorption behavior has further verified the hypothesis we proposed when designing this catalyst: M sites in the Cr‐NiCoP_v_@NF electrocatalyst belong to hard LAs, and OH^−^ ions belong to harder LBs than Cl^−^ions. According to the theory of HSAB, this enables the M sites to preferentially absorb OH^−^ and form a molecular‐level protective shield, thereby preventing the attack of Cl^−^ through electrostatic repulsion, while the P sites, as LB sites, have no approximate affinity for Cl^−^ since chloride ions also belong to Lewis bases.^[^ ^29^, ^38^, ^39^ ^]^ The synergy between these effects creates an ideal environment for stable HER operation in corrosive seawater conditions. This mechanism provides a theoretical basis for the catalyst to present excellent stability under intermittent operation conditions, enabling the catalyst to maintain outstanding performance continuously in multiple start‐shutdown cycles. This dynamic protection mechanism represents a significant advancement over conventional corrosion‐resistant designs that rely on passive coatings or completely inert materials.

It should be noted that although the absolute values of the adsorption energy and reaction energy barrier are affected by the choice of computational model, the key comparative conclusion is that the robust use of implicit solvation and computational hydrogen electrode models provides a reliable framework for evaluating the relative trends of catalytic activity and ion selectivity despite surface termination methods Changes in P‐vacancy density or adsorbate coverage may quantitatively shift the energy landscape, but they are not expected to alter the fundamental trend of enhanced HER kinetics and resistance to chloride ion corrosion mediated by FLP sites. In summary, the theoretical calculations elucidated the origin of the excellent HER activity of Cr‐NiCoP_v_@NF from three dimensions: electronic structure modulation, energy barrier optimization, and interfacial adsorption properties, and provided the theoretical basis for the corrosion resistance exhibited in the shutdown stage.

### 2.4 Performance of Intermittent Alkaline Seawater Electrolysis

**Figure 5:**
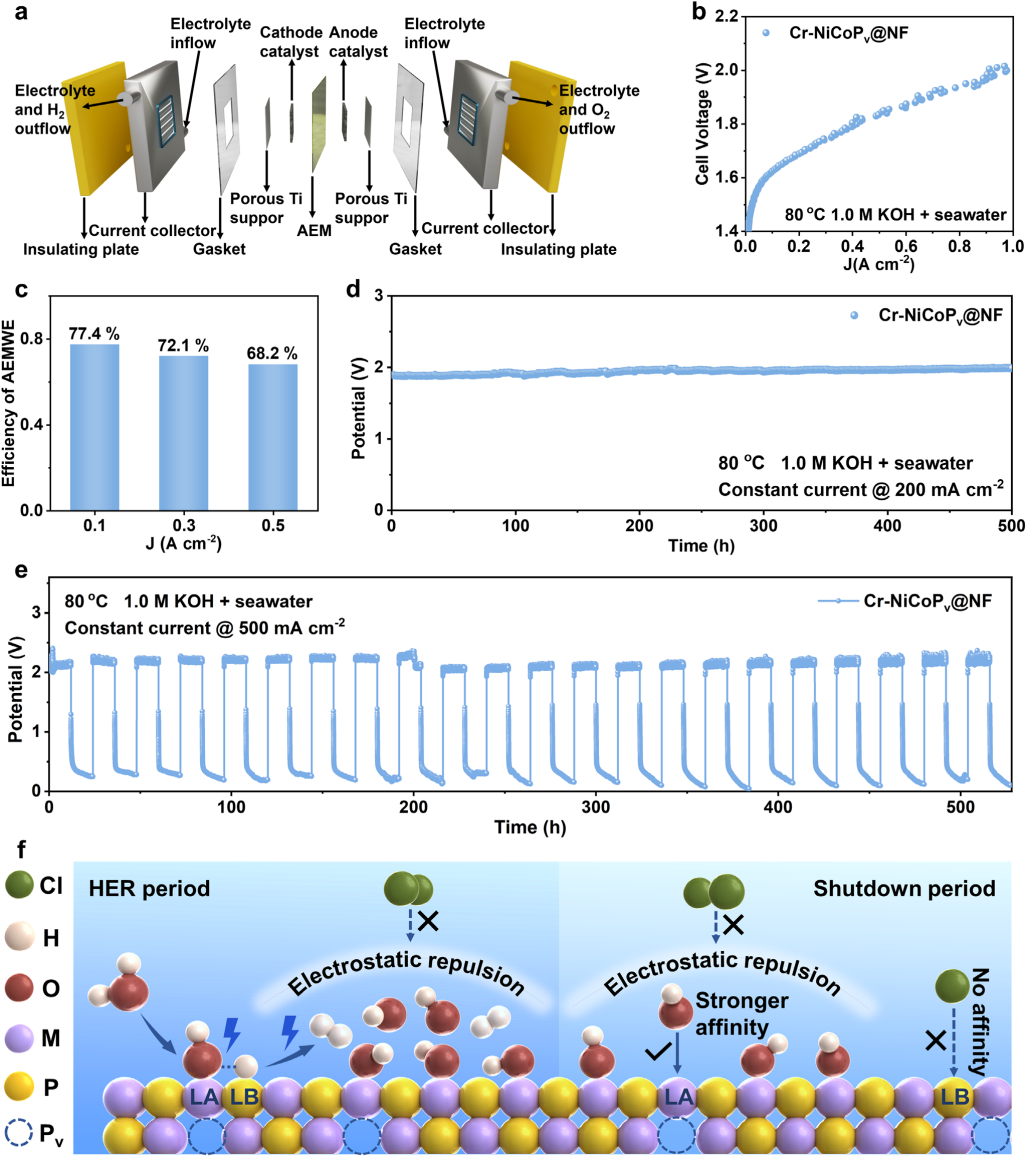
AEM electrolyzer performance evaluation. a) Model diagram of an anion exchange membrane electrolyzer. b) Polarization curve in the AEM electrolyzer using Cr‐NiCoP_v_@NF as cathode and NiFe LDH as anode. c) Efficiency of the AEM electrolyzer at different current densities. d) Chronopotentiometry curve in the AEM electrolyzer using Cr‐NiCoP_v_@NF as cathode and NiFe LDH as anode operated at 0.2 A cm^−2^ in 1.0 m KOH + seawater electrolyte at 80 °C. e) Intermittent stability test in the AEM electrolyzer (with Cr‐NiCoP_v_@NF cathode and NiFeP anode) recorded at 0.5 A cm^−2^ in 1.0 m KOH + seawater electrolyte with 12 h start and 12 h shutdown cycles at 80 °C. f) Schematic diagram of Cr‐NiCoP_v_@NF electrocatalyst for accelerating HER and repelling chloride ions.

To assess the industrial viability of the Cr‐NiCoP_v_@NF cathode catalyst for seawater electrolysis, we engineered an anion‐exchange membrane water electrolyzer (AEMWE) system employing Cr‐NiCoP_v_@NF as the cathode paired with a NiFe LDH anode. **Figure** **5**a schematically illustrates the structural architecture and operational mechanism of the AEM electrolyzer employed in this study. The detailed textual descriptions of this device are provided in Note S1 (Supporting Information). The electrolyzer demonstrated outstanding electrochemical performance in 1.0 m KOH + seawater electrolyte at 80 °C. Polarization curve analysis revealed that the system requires only 2.0 V operating voltage to achieve a current density of 1 A cm^−2^, enabling higher current density at lower voltage, reducing energy consumption, and improving efficiency (Figure 5b). Energy conversion efficiency represents a paramount metric for industrial hydrogen production systems. AEMWE efficiency calculations demonstrated a remarkable energy conversion efficiency of 77.4% at 100 mA cm^−2^ (Figure 5c). This exceptional efficiency will directly translate to substantial economic benefits, as confirmed by the techno‐economic analysis. A simple economic evaluation indicated that when only the efficiency of AEM electrolyzers is considered without taking into account investment costs such as equipment and labor, the cost of hydrogen production is $0.87 per gasoline gallon equivalent (GGE) H_2_ (Note S2, Supporting Information). This value is significantly lower than the U.S. Department of Energy's 2026 target of $2.00 per GGE H_2_, highlighting the exceptional cost‐effectiveness of this catalytic system for industrial‐scale hydrogen production. This cost advantage is further enhanced by the anticipated reduction in renewable electricity prices. The stability and durability of electrolysis systems under continuous operation represent equally critical factors for industrial adoption. Under industrial operating conditions (200 mA cm^−2^, 80 °C, 1 m KOH + seawater electrolyte), the electrolyzer demonstrated exceptional operational stability, maintaining consistent performance for over 500 h with a stable cell voltage of ≈1.9 V with less than 0.22 mV h^−1^ activity decay (Figures 5d; S21, Supporting Information).

The intermittency of renewable electricity sources is a critical consideration for the practical implementation of seawater electrolysis. Systematic chronopotentiometry investigations revealed critical voltage reversal phenomena during shutdown phases, with the cathode potential reaching as high as 1.0 V versus reversible hydrogen electrode (RHE) after 100 min of system shutdown (Figure S22, Supporting Information). These voltage inversions pose severe thermodynamic conditions that can cause irreversible oxidative damage to the cathode catalyst. This issue can be exacerbated when seawater is used as a feedstock, as halide anions tend to accumulate at the cathode during shutdown, leading to cathode and current collector corrosion.^[^ ^9^ ^]^ This preliminary finding highlights the necessity for robust catalyst design that can withstand such extreme electrochemical conditions while maintaining structural and functional integrity.

A key conceptual advance of this work is evaluating electrocatalyst compatibility with intermittent renewable electricity, a critical requirement for practical green hydrogen production yet rarely integrated into catalyst design. We designed a demanding 12 h start‐shutdown cycling protocol to simulate the inherent volatility of solar/wind power generation, incorporating both operational periods and complete shutdowns to represent day‐night cycle‐induced fluctuations. Although the cathode maintained excellent performance throughout the entire testing process, initial testing with the Cr‐NiCoP_v_@NF cathode and NiFe LDH anode revealed system instability, manifested by abnormally high voltage on day 4 (Figure S23, Supporting Information). Post‐test analysis identified severe anode corrosion (with only 2/3 material remaining), while the cathode maintained macroscopic structural integrity (Figure S24, Supporting Information). Subsequently, a 12 h start‐shutdown cycle test was conducted in 1.0 m KOH + seawater electrolyte at 80 °C using more corrosion‐resistant NiFeP anodes, which prevented premature system failure due to anode degradation (Figure 5e). During the comprehensive 520 h cycle durability testing period, the Cr‐NiCoP_v_@NF material demonstrated exceptional cycling consistency, with nearly identical potential fluctuation patterns in each cycle, showing neither significant attenuation nor deviation in performance. XPS and Raman characterizations confirm that a slight reconstruction occurred on the Cr‐NiCoP_v_@NF surface after the long‐time intermittent test (Figures S25 and S26, Supporting Information). This outstanding cyclic stability suggests that the FLP‐based material can maintain its electrochemical properties with minimal degradation under long‐term intermittent operation. Chronopotentiometry curve using Cr‐NiCoP_v_@NF as anode further confirms Cr‐NiCoP_v_@NF electrode's excellent chloride ion repellency during the voltage reversal occurring at the shutdown stage (Figure S27, Supporting Information). The lower concentration of hypochlorite detected with Cr‐NiCoP_v_@NF, as compared to NiCoP_v_@NF, serves as direct evidence of its superior durability and enhanced resistance to chloride oxidation (Figure S28, Supporting Information). Inductively coupled plasma‐Mass Spectrometry (ICP‐MS) testing proved that the dissolution amount of Cr in the electrolyte was trace after a long‐time long‐term intermittent operation, highlighting its robust structural integrity and electrochemical durability (Table S2, Supporting Information). This stability is crucial for practical seawater electrolysis applications, especially in harsh operating environments that require continuous performance under intermittent start‐shutdown and other adverse conditions. Cr‐NiCoP_v_@NF material can resist performance degradation during long‐term operation, making it a very promising candidate for industrial‐scale applications where reliability and lifespan are of critical importance. These stability properties provide convincing experimental validation for the practical industrial application of Cr‐NiCoP_v_@NF materials.

The Cr‐NiCoP_v_@NF self‐supported electrode delivers industrial‐current‐density HER activity surpassing typical non‐precious metal catalysts, alongside corrosion resistance and sustained operation exceeding 520 h under simulated real‐world conditions. These results position it as a promising alternative to precious metal‐based catalysts. The excellent performance stems from its unique FLP structure, which simultaneously addresses the dual key challenges in industrial electrolysis (Figure 5f): 1) The H_2_O dissociation energy barrier and ^*^H desorption energy barrier have been reduced by an electron “tug‐of‐war” effect of the FLP site, enabling the catalyst to maintain high activity at high current densities. 2) During HER operation, the high OH^−^ local concentration on the electrocatalyst surface generated by water dissociation blocks Cl^−^ access to the vulnerable FLP active sites. During shutdown periods, the preferential adsorption of OH^−^ onto the Lewis acid sites as dictated by HSAB theory, forms a molecular‐scale protective layer. The collective action of this layer and the Lewis base sites creates a negatively charged surface, thereby continuously repelling approaching Cl^−^ ions through electrostatic repulsion. Since OH^−^ preferential adsorption is thermodynamically driven, this protective layer can be maintained dynamically, rather than being a transient process, as long as the surface LA sites are present. This mechanism ensures exceptional long‐term stability under intermittent operation. Our work represents a significant advancement in seawater electrolysis technology, with the Cr‐NiCoP_v_@NF catalyst demonstrating necessary characteristics for commercial implementation: high efficiency, exceptional durability, and competitive economics. The successful integration of this catalyst into a complete AEMWE system, coupled with its proven compatibility with intermittent operation, positions it as a leading candidate to replace precious‐metal catalysts in next‐generation electrolyzers. These achievements establish a viable pathway for cost‐competitive green hydrogen production from abundant seawater resources, addressing critical challenges in the transition to sustainable energy systems. The fundamental design principles demonstrated here, particularly the FLP‐mediated dual functionality, provide a valuable framework for future catalyst development targeting industrial‐scale electrochemical applications.

## 3 Discussion

Our work establishes a FLP design paradigm for seawater electrolysis, resolving the persistent trade‐off between catalytic activity and dynamic stability in non‐precious cathodes. The Cr‐NiCoP_v_@NF catalyst achieves a HER overpotential of 110 mV at 10 mA cm^−2^ in alkaline seawater electrolysis, representing a 51.1% reduction versus NiCo LDH@NF non‐FLP benchmarks, and sustains operation for over 520 h under intermittent renewable power simulations. Through integrated theoretical and experimental analysis, we elucidate the FLP bifunctional mechanism: 1) Dual energetic optimization lowers the water dissociation barrier to 0.50 eV while tuning ^*^H desorption to 0.58 eV, circumventing alkaline HER kinetics limits; 2) Anion selective adsorption generates a molecular‐level barrier against electrocatalyst corrosion, where stronger OH^−^ affinity (−3.26 eV) than Cl^−^ (−1.79 eV). By unifying high activity and corrosion resistance, this FLP strategy provides a generalizable framework for industrial‐scale green hydrogen production from seawater.

## Conflict of Interest

The authors declare no conflict of interest.

## Supporting information



Supporting Information

## Data Availability

The data that support the findings of this study are available from the corresponding author upon reasonable request.
