# Supplementary material for: Dynamic Stability in Intermittent Seawater Electrolysis Via Frustrated Lewis Pair Engineering
Source: Adv Sci (Weinh). 2025 Nov 25;13(7):e18514. doi: 10.1002/advs.202518514 (PMC12866868; doi:10.1002/advs.202518514)
Supplement: Supplementary file 1 — Supporting Information [file ADVS-13-e18514-s001.docx]

**Dynamic Stability in Intermittent Seawater Electrolysis via Frustrated Lewis Pair Engineering**

Peilin Shen ^a†^, Jiawei Zhu ^a†^, Chen Deng ^b, c*^, Shangqian Zhu ^b^, Xiaoman He ^a^, Wenguang Ouyang ^b^, Xin Tu ^c^, Huiyan Zhang ^a*^, Richen Lin ^a*^

^a^ Key Laboratory of Energy Thermal Conversion and Control of Ministry of Education, School of Energy and Environment, Southeast University, Nanjing 211189, China

^b^ School of Chemistry and Chemical Engineering, Southeast University, Nanjing 211189, China

^c^ Department of Electrical Engineering and Electronics, University of Liverpool, Liverpool L69 3GJ, UK

^†^ These authors contributed equally to this work.

* Corresponding authors.

E-mail: chen.deng2@liverpool.ac.uk (C. Deng); hyzhang@seu.edu.cn (H. Zhang); richenlin@seu.edu.cn (R. Lin)

**Part I. Experimental methods**

**Materials**

Nickel nitrate hexahydrate (Ni(NO_3_)_2_·6H_2_O, AR), Cobalt nitrate hexahydrate (Co(NO_3_)_2_·6H_2_O, AR), chromium nitrate nine-hydrate (Cr(NO_3_)_3_·9H_2_O, AR), ammonium fluoride (NH_4_F, 96.0%), urea (CH_4_N_2_O, AR), sodium monophosphate (NaPO_2_H_2_·H_2_O, 99%), potassium hydroxide (KOH, AR), Hydrochloric acid (HCl, 0.1 mol L^-1^), ethanol (C_2_H_5_OH, AR), deionized water (DI), seawater was taken from the Yellow Sea, China, nickel foam (NF, 1.0 mm thickness).

**Synthesis of Cr-NiCo LDH@NF**

Cr-NiCo LDH@NF was synthesized on nickel foam through a hydrothermal method. In a typical synthesis, a piece of commercial Ni foam (2 × 3 cm^2^) was cleaned by ultrasonication with 0.1 M diluted hydrochloric acid solution and ethanol for 30 min, respectively, and then rinsed with deionized water and ethanol sequentially to remove impurities from the NF surface. The pre-treated nickel foam was then transferred into a Teflon-lined stainless-steel autoclave (50 ml) containing a homogenous solution of 30 ml deionized water, Ni(NO_3_)_2_·6H_2_O (1 mmol), Co(NO_3_)_2_·6H_2_O (1 mmol), Cr(NO_3_)_3_·9H_2_O (0.3 mmol), NH_4_F (2.5 mmol) and urea (CH_4_N_2_O, 6 mmol). Afterwards, the autoclave was sealed and maintained at 120 °C for 6 h. After reaction, the sample was taken out and washed three times with deionized water and ethanol, respectively, and then dried at 60 °C for 10 h under vacuum.

**Synthesis of Cr-NiCoP_v_@NF**

Cr-NiCoP_v_@NF was synthesized by phosphorization of Cr-NiCo LDH@NF in a tube furnace. Briefly, 0.5 g of NaH_2_PO_2_·H_2_O was placed upstream in a quartz tube to serve as the phosphorous source, whereas the obtained Cr-NiCo LDH@NF was placed at the centre of the quartz tube, and then the quartz tube was heated at 350 °C for 2 h with a heating rate of 4 °C min^-1^ in an Ar atmosphere. After reaction, the furnace was turned off and naturally cooled to room temperature under an Ar atmosphere to obtain Cr-NiCoP_v_@NF. The amount of NaH_2_PO_2_·H_2_O depends on the Cr-NiCo LDH@NF surface area; specifically, 0.5 g of NaH_2_PO_2_·H_2_O corresponds to a Cr-NiCo LDH@NF area of 4 cm^2^.

**Synthesis of NiCo LDH@NF**

NiCo LDH@NF was synthesized on nickel foam through a hydrothermal method. In a typical synthesis, a piece of commercial Ni foam (2 × 3 cm^2^) was cleaned by ultrasonication with 0.1 M diluted hydrochloric acid solution and ethanol for 30 min, respectively, and then rinsed with deionized water and ethanol sequentially to remove impurities from the NF surface. The pre-treated nickel foam was then transferred into a Teflon-lined stainless-steel autoclave (50 ml) containing a homogenous solution of 30 ml deionized water, Ni(NO_3_)_2_·6H_2_O (1 mmol), Co(NO_3_)_2_·6H_2_O (1 mmol), NH_4_F (2.5 mmol), and urea (CH_4_N_2_O, 6 mmol). Afterwards, the autoclave was sealed and maintained at 120 °C for 6 h. After reaction, the sample was taken out and washed three times with deionized water and ethanol, respectively, and dried at 60 °C for 10 h under vacuum.

**Synthesis of NiCoP_v_@NF**

NiCoP_v_@NF was synthesized by phosphorization of NiCo LDH@NF in a tube furnace. Briefly, 0.5 g of NaH_2_PO_2_·H_2_O was placed upstream in a quartz tube to serve as the phosphorous source, whereas the obtained NiCo LDH@NF was placed at the centre of the quartz tube, and then the quartz tube was heated at 350 °C for 2 h with a heating rate of 4 °C min^-1^ in an Ar atmosphere. After reaction, the furnace was turned off and naturally cooled to room temperature under an Ar atmosphere to obtain NiCoP_v_@NF. The amount of NaH_2_PO_2_·H_2_O depends on the NiCo LDH@NF surface area; specifically, 0.5 g of NaH_2_PO_2_·H_2_O corresponds to a NiCo LDH@NF area of 4 cm^2^.

**Synthesis of NiFe LDH**

NiFe LDH was synthesized on nickel foam through a soaking method. In a typical synthesis, a piece of commercial Ni foam (2 × 2 cm^2^) was cleaned by ultrasonication with 0.1 M diluted hydrochloric acid solution and ethanol for 30 min, respectively, and then rinsed with deionized water and ethanol sequentially to remove impurities from the NF surface. The pre-treated nickel foam was then transferred into a beaker (50 ml) containing a homogenous solution of 20 ml deionized water and Fe(NO_3_)_3_·9H_2_O (2 mmol). Afterwards, the beaker was maintained at 34 °C for 2 h. After reaction, the sample was taken out and washed three times with deionized water and ethanol, respectively, and dried at 60 °C for 1 h.

**Synthesis of NiFeP**

NiFe LDH-ht was synthesized on nickel foam through a hydrothermal method. In a typical synthesis, a piece of commercial Ni foam (2 × 4 cm^2^) was cleaned by ultrasonication with 0.1 M diluted hydrochloric acid solution and ethanol for 30 min, respectively, and then rinsed with deionized water and ethanol sequentially to remove impurities from the NF surface. The pre-treated nickel foam was then transferred into a Teflon-lined stainless-steel autoclave (50 ml) containing a homogenous solution of 35 ml deionized water, Ni(NO_3_)_2_·6H_2_O (1 mmol), Fe(NO_3_)_3_·9H_2_O (1 mmol), NH_4_F (2.5 mmol), and urea (CH_4_N_2_O, 6 mmol). Afterwards, the autoclave was sealed and maintained at 120 °C for 6 h. After reaction, the sample was taken out and washed three times with deionized water and ethanol, respectively, and then dried at 60 °C for 10 h under vacuum.

NiFeP was synthesized by phosphorization of NiFe LDH-ht in a tube furnace. Briefly, 0.5 g of NaH_2_PO_2_·H_2_O was placed upstream in a quartz tube to serve as the phosphorous source, whereas the obtained NiFe LDH-ht was placed at the centre of the quartz tube, and then the quartz tube was heated at 350 °C for 2 h with a heating rate of 4 °C min^-1^ in an Ar atmosphere. After reaction, the furnace was turned off and naturally cooled to room temperature under an Ar atmosphere to obtain NiFeP. The amount of NaH_2_PO_2_·H_2_O depends on the NiFe LDH-ht surface area; specifically, 0.5 g of NaH_2_PO_2_·H_2_O corresponds to a NiFe LDH-ht area of 4 cm^2^.

**Pretreatment methods for seawater electrolyte**

First, an appropriate amount of KOH solid was added to the raw seawater collected from the Yellow Sea, China, to prepare a mixed electrolyte with a final concentration of 1.0 M KOH + seawater. The alkalized solution was allowed to stand thoroughly until the precipitates completely settled, and the supernatant was taken as the experimental electrolyte. The prepared supernatant electrolyte was stored at room temperature in a sealed condition to avoid interference from substances such as carbon dioxide in the air.

**Materials Characterizations**

The morphology and elemental distribution of the samples were analyzed by scanning electron microscopy (SEM) (ZEISS Sigma 360, Germany) and transmission electron microscopy (TEM) (FEI TalosF200x). The phase composition of the catalyst was characterised with an X-ray diffraction (XRD) (Rigaku SmartLab SE, Japan) instrument at a sweep speed of 5°min^-1^ and 2θ range of 5 ~ 90°. The presence of phosphorus vacancies was detected by electron paramagnetic resonance (EPR) (Bruker A300) spectroscopy. The elemental valence states on the sample surface were determined by X-ray photoelectron spectroscopy (XPS) (Shimadzu/Krayos AXIS Ultra DLD, Japan). All binding energies were referenced to the C 1s peak (284.8 eV). CO_2_ and NH_3_ temperature programmed desorption (TPD) were performed on a BUILD PCA-1200 chemisorption analyser. In situ Raman analysis was performed on the Raman instrument (XploRA PLUS) using a 50x objective lens. The laser power is 100 mW, and the wavelength is 473 nm. It takes 20 seconds to obtain one spectrum each time.

Ni K-edge analysis was performed with Si (111) crystal monochromators at the BL11B beamlines at the Shanghai Synchrotron Radiation Facility (SSRF) (Shanghai, China). Before the analysis at the beamline, samples were pressed into thin sheets with a diameter of 1 cm in diameter and sealed using Kapton tape film. The XAFS spectra were recorded at room temperature using a 4-channel Silicon Drift Detector (SDD) Bruker 5040. Ni K-edge extended X-ray absorption fine structure (EXAFS) spectra were recorded in transmission mode. Negligible changes in the line shape and peak position of Ni K-edge XANES spectra were observed between two scans taken for a specific sample. The spectra were processed and analysed by the software codes Athena and Artemis.

**Electrochemical Measurement**

Except for the cyclic voltammetry test, which uses a CHI 660E electrochemical workstation and the long-term stability test, which uses a Gamry Reference 3000 electrochemical workstation, all other electrochemical measurements were executed using an Ivium-N-stat electrochemical workstation. Hg/HgO and graphite electrodes were employed as reference and control electrodes, respectively. The NF was utilized as a self-supported material to fabricate all the catalysts. In these experiments, the potential was calibrated according to the reversible hydrogen electrode (RHE) as follows: E(RHE) = E(Hg/HgO) + 0.098 V + 0.059pH. Hydrogen evolution reaction (HER) tests were conducted in solutions of 1.0 M KOH and 1.0 M KOH + seawater (after removing the sediment). Linear scanning voltammetry (LSV) polarization curves were generated at a scan rate of 10 mV s^-1^. All LSV curves have undergone iR-compensation unless otherwise stated. Electrochemical impedance spectra (EIS) were recorded by applying an alternating current voltage of 5 mV within the frequency range of 0.1 ~ 10^5^ Hz. Electrochemically active surface area measurements were acquired by conducting cyclic voltammetry (CV) cycles at various scan rates (40 to 120 mV s^-1^). The electrochemically active surface area (ECSA) was determined from the double-layer capacitance (C_dl_) measured by CV. The specific ECSA was calculated using the following equation: ECSA = C_dl_/C_s_, where C_s_ is the specific capacitance of an atomically smooth planar surface under identical electrolyte conditions. Based on previously reported literature^[1, 2]^, the usual specific capacitance of C_s_ = 40 μF cm^-2^ was used to evaluate ECSA in this work. Long-term stability and intermittent stability assessments were performed at 80°C utilizing the chronopotentiometry (V-t) method without iR-compensation.

**Density functional theory (DFT) calculations**

All the calculations are performed in the framework of the density functional theory with the projector augmented plane-wave method, as implemented in the Vienna ab initio simulation package^[3]^. The generalized gradient approximation proposed by Perdew, Burke, and Ernzerhof is selected for the exchange-correlation potential^[4]^. The long-range van der Waals interaction is described by the DFT-D3 approach^[5]^. The cut-off energy for the plane wave is set to 500 eV. The energy criterion is set to 10^-5^ eV in the iterative solution of the Kohn-Sham equation. A vacuum layer of 15 Å is added perpendicular to the sheet to avoid artificial interaction between periodic images. The Brillouin zone integration is performed using a 2 × 2 × 1 k-mesh. All the structures are relaxed until the residual forces on the atoms have declined to less than 0.05 eV Å^-1^.

**Part II. Supplementary figures, tables, and notes**

**
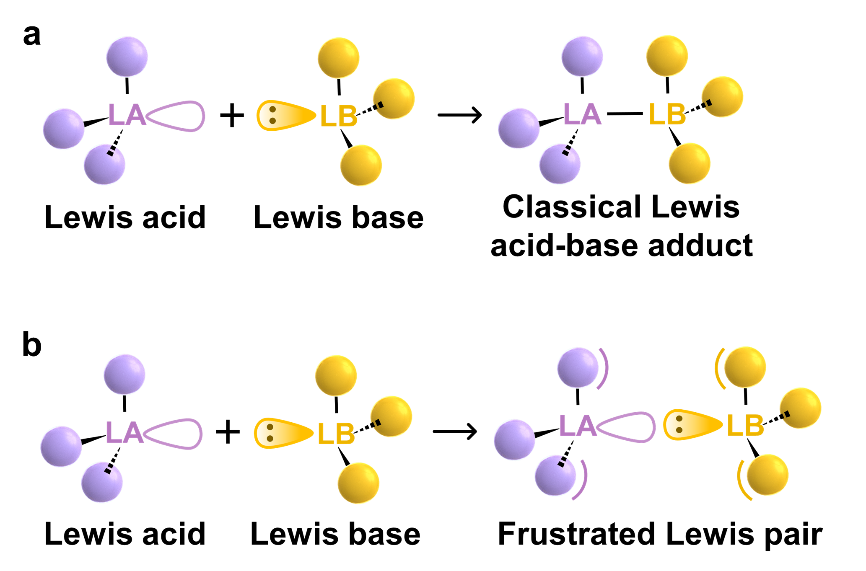
**

**Supplementary Figure 1.** (a) Classical Lewis acid-base adduct. (b) Frustrated Lewis pair.

**
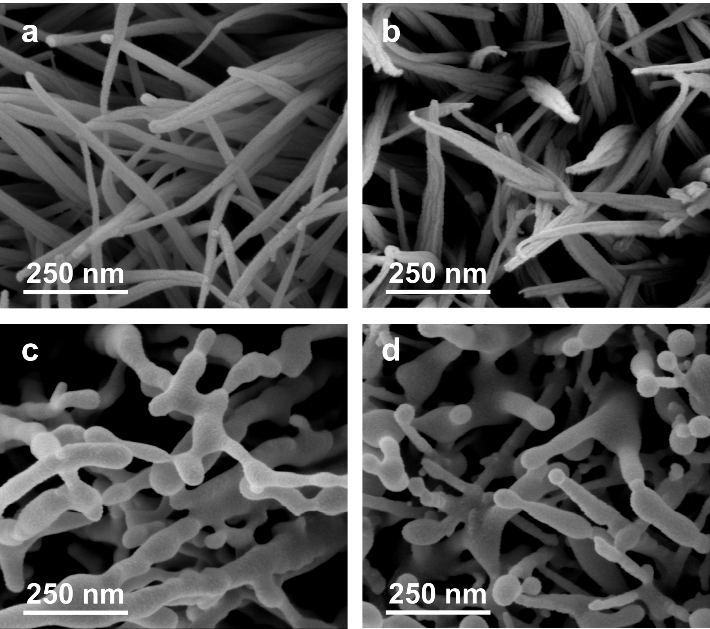
**

**Supplementary Figure 2.** SEM images of the (a) NiCo LDH@NF, (b) Cr-NiCo LDH@NF, (c) NiCoP_v_@NF, and (d) Cr-NiCoP_v_@NF


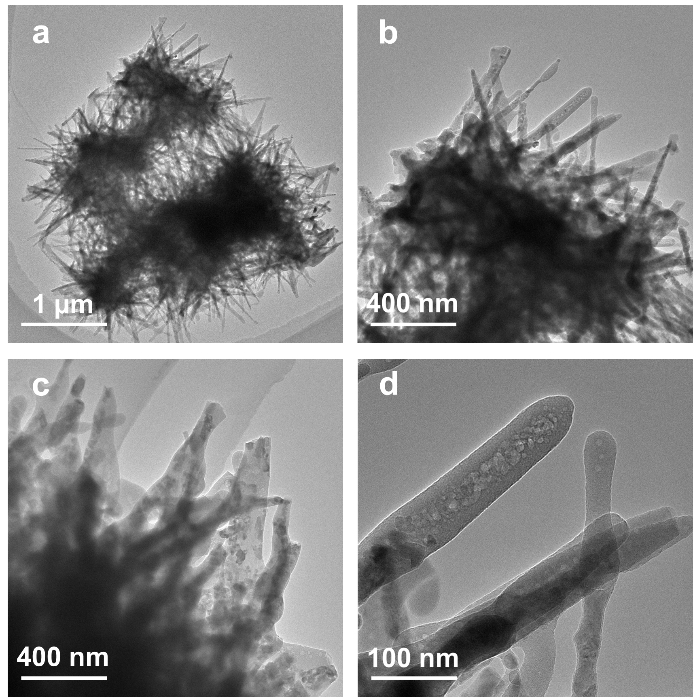


**Supplementary Figure 3.** TEM images of the Cr-NiCoP_v_@NF at different scales.

**
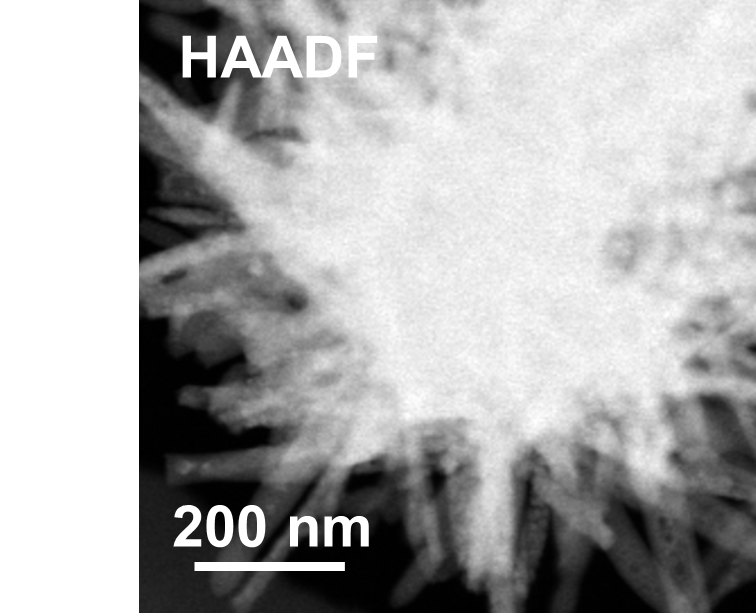
**

**Supplementary Figure 4.** High-Angle Annular Dark-Field (HAADF) of the Cr-NiCoP_v_@NF.


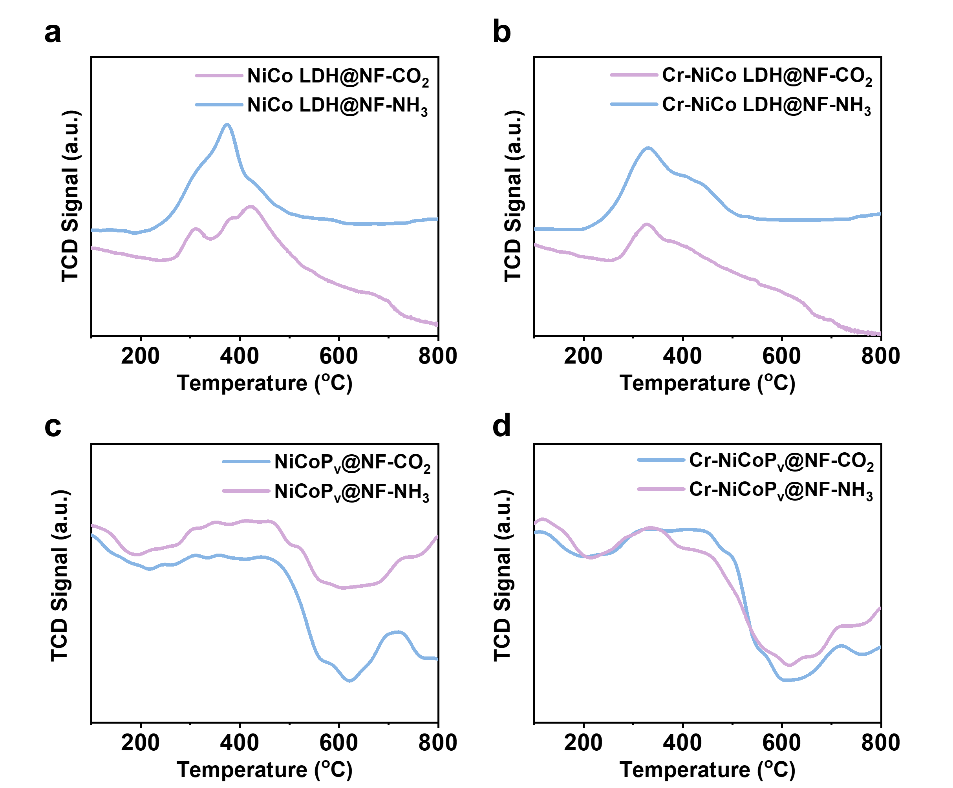


**Supplementary Figure 5.**  NH_3_-TPD and CO_2_-TPD curve of the (a) NiCo LDH@NF, (b) Cr-NiCo LDH@NF, (c) NiCoP_v_@NF, and (d) Cr-NiCoP_v_@NF.


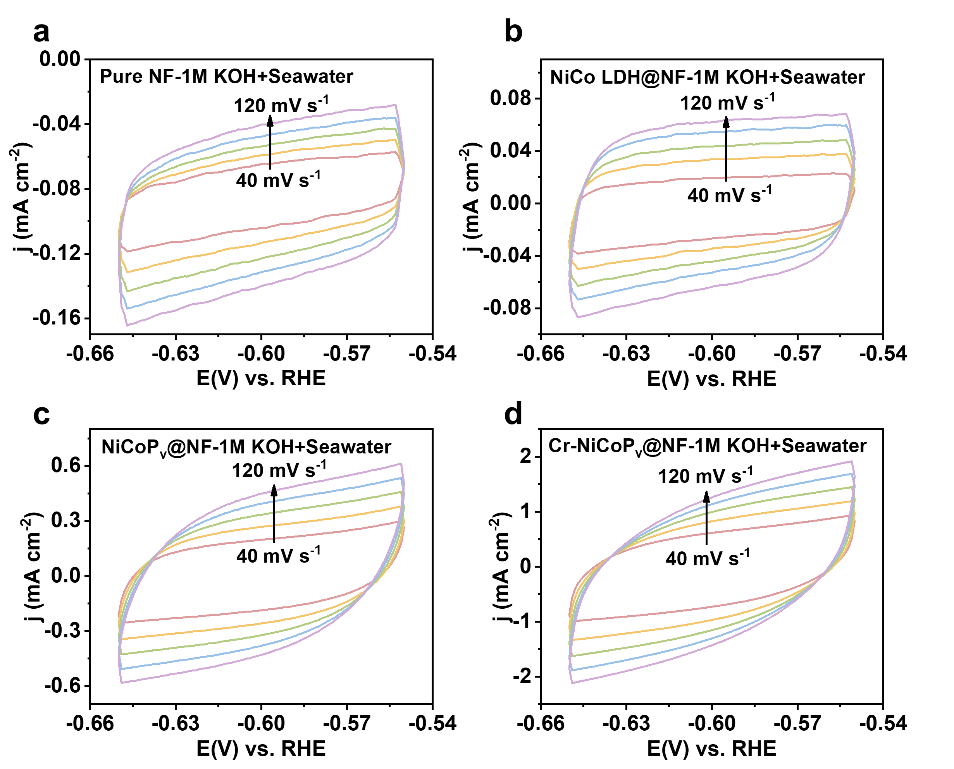


**Supplementary Figure 6.** Cyclic voltammetry curves of the (a) NiCo LDH@NF, (b) Cr-NiCo LDH@NF, (c) NiCoP_v_@NF, and (d) Cr-NiCoP_v_@NF in 1.0 M KOH + seawater electrolyte.


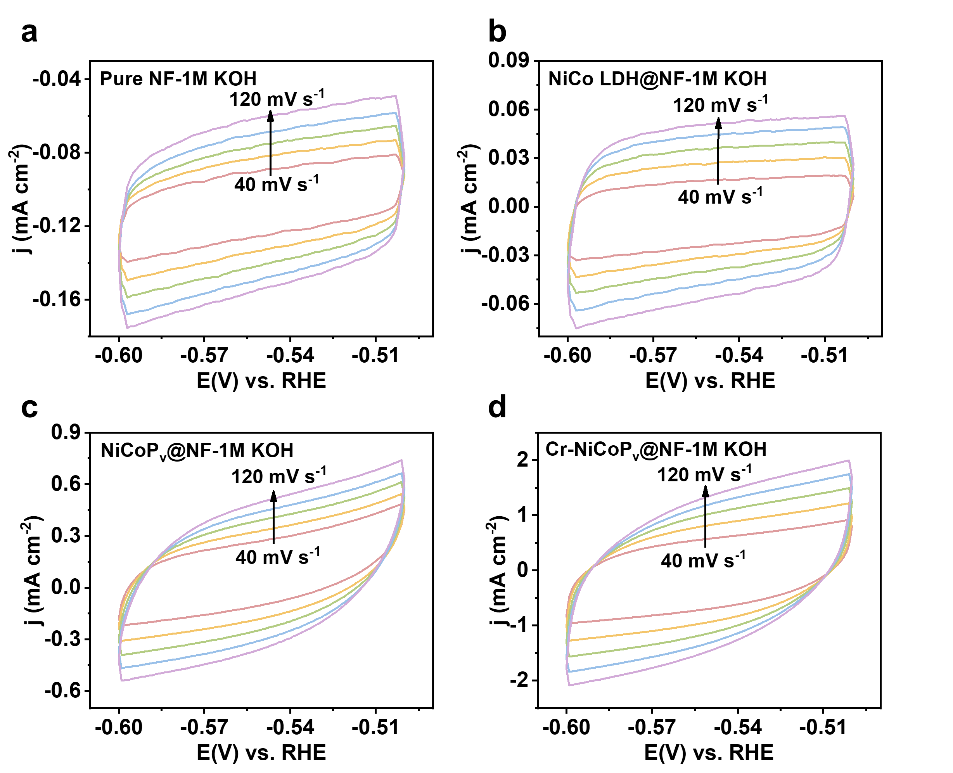


**Supplementary Figure 7.** Cyclic voltammetry curves of the (a) NiCo LDH@NF, (b) Cr-NiCo LDH@NF, (c) NiCoP_v_@NF, and (d) Cr-NiCoP_v_@NF in 1.0 M KOH electrolyte.

**
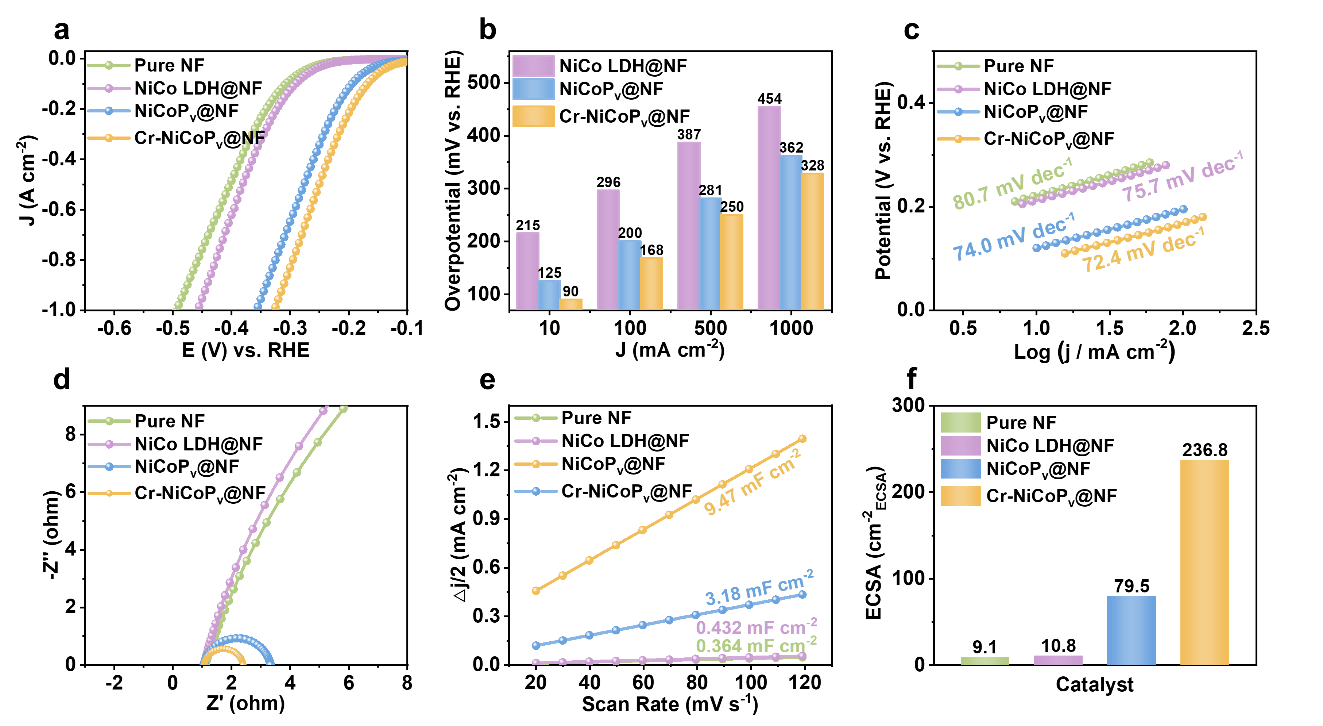
**

**Supplementary Figure 8. Electrochemical performance of the Cr-NiCoP_v_@NF and control samples in 1.0 M KOH electrolyte.** (a) Representative HER polarization curves. (b) The overpotential at a current density of 0.01, 0.1, 0.5, and 1 A cm^-2^. (c) Tafel slopes. (d) Nyquist plots. (e) Double-layer capacitances. (f) Electrochemically active surface area.


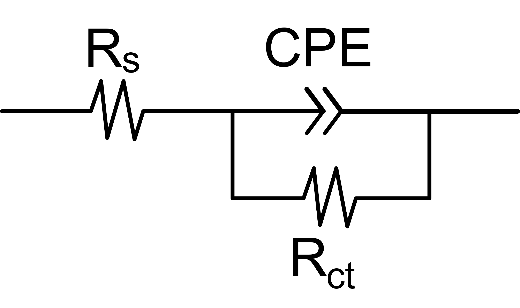


**Supplementary Figure 9.** Equivalent circuit model of EIS.


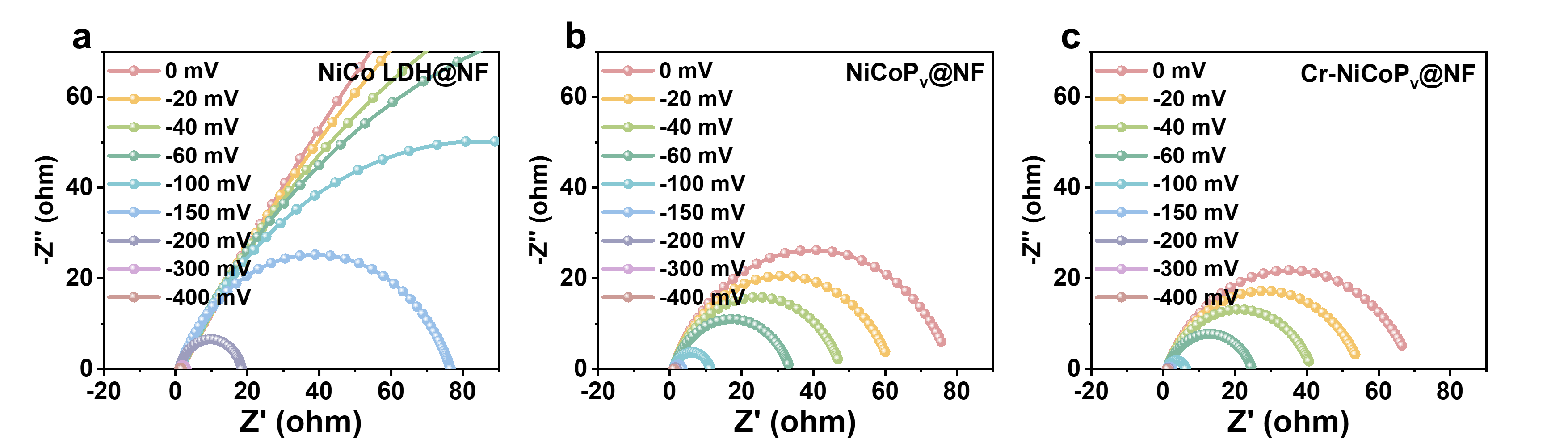


**Supplementary Figure 10.** In situ Nyquist plots of the (a) NiCo LDH@NF, (b) NiCoP_v_@NF, and (c) Cr-NiCoP_v_@NF at different applied overpotentials in 1.0 M KOH electrolyte.


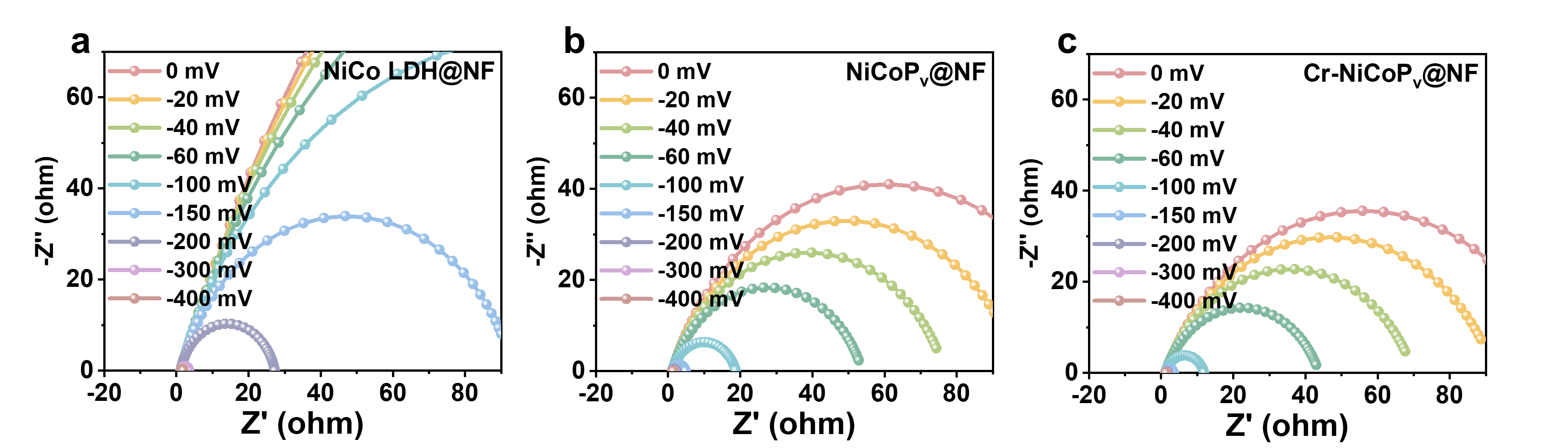


**Supplementary Figure 11.** In situ Nyquist plots of the (a) NiCo LDH@NF, (b) NiCoP_v_@NF, and (c) Cr-NiCoP_v_@NF at different applied overpotentials in 1.0 M KOH + seawater electrolyte.


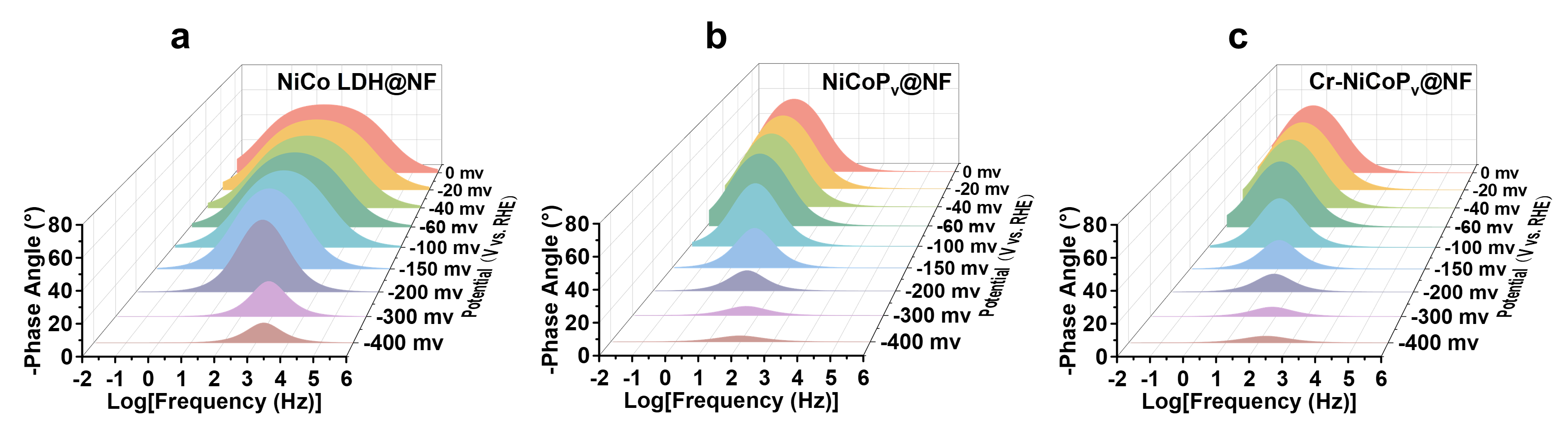


**Supplementary Figure 12** In situ Bode phase plots of the (a) NiCo LDH@NF, (b)NiCoP_v_@NF, and (c) Cr-NiCoP_v_@NF at different applied overpotentials in 1.0 M KOH electrolyte.

**Supplementary Figure 13.** In situ Bode phase plots of the NiCoP_v_@NF at different applied overpotentials in 1.0 M KOH + seawater electrolyte.

**
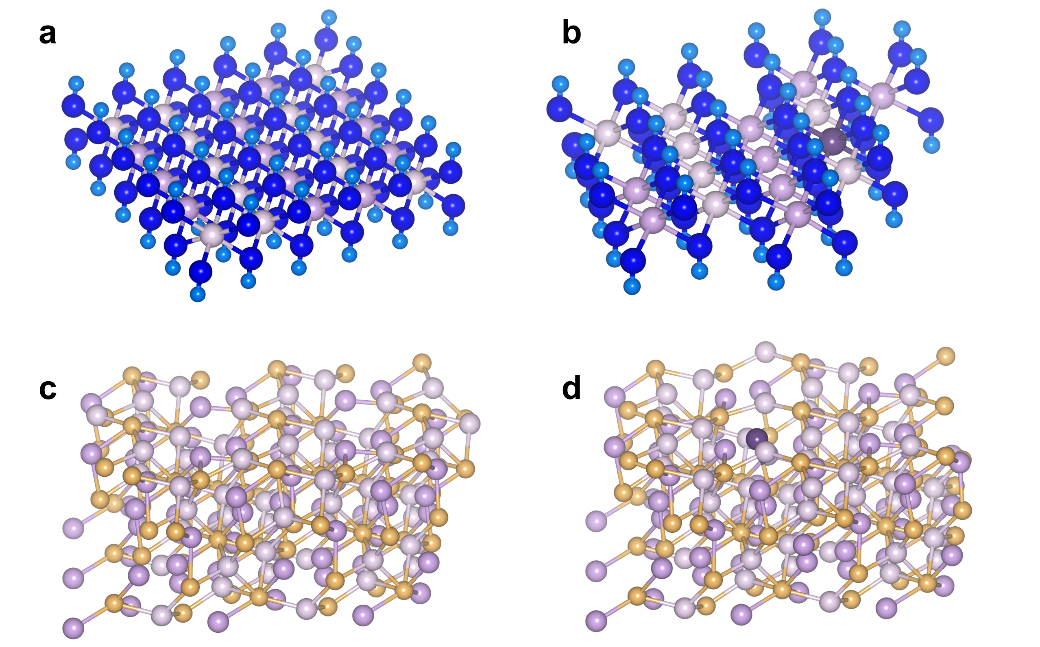
**

**Supplementary Figure 14.** Theoretical model for DFT calculation of the (a) NiCo LDH@NF, (b) Cr-NiCo LDH@NF, (c) NiCoP_v_@NF, and (d) Cr-NiCoP_v_@NF.


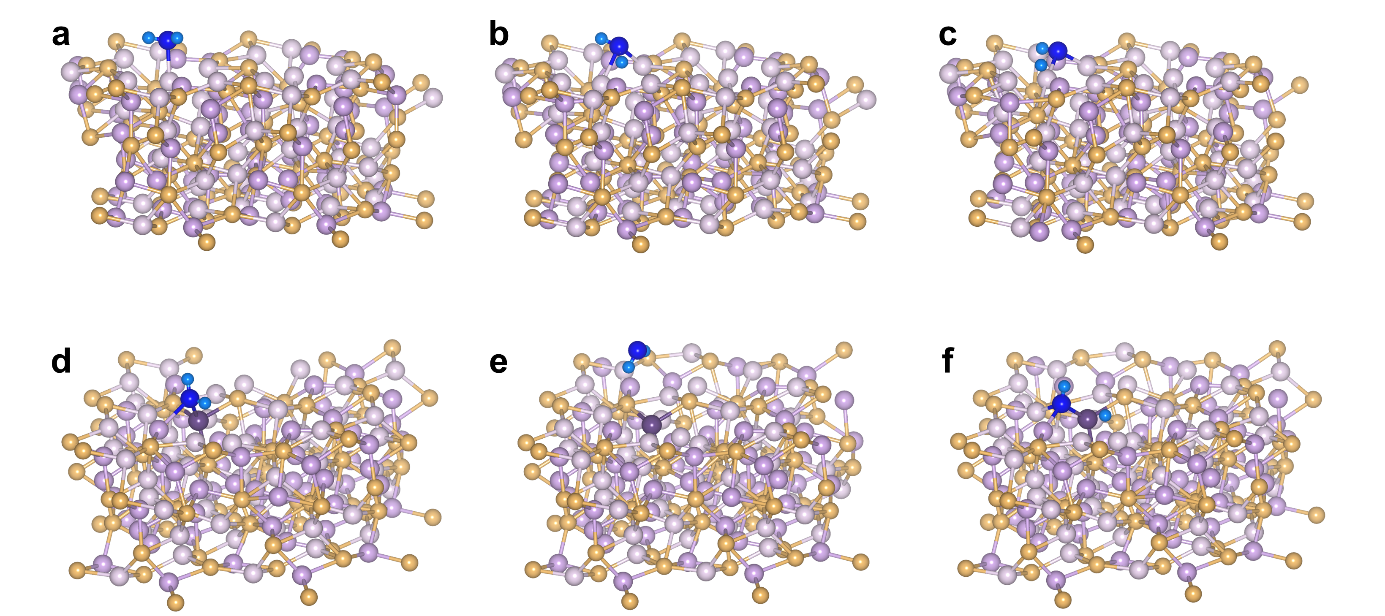


**Supplementary Figure 15.** Theoretical model for DFT calculation of water dissociation process of the (a-c) NiCoP_v_@NF and (d-f) Cr-NiCoP_v_@NF.


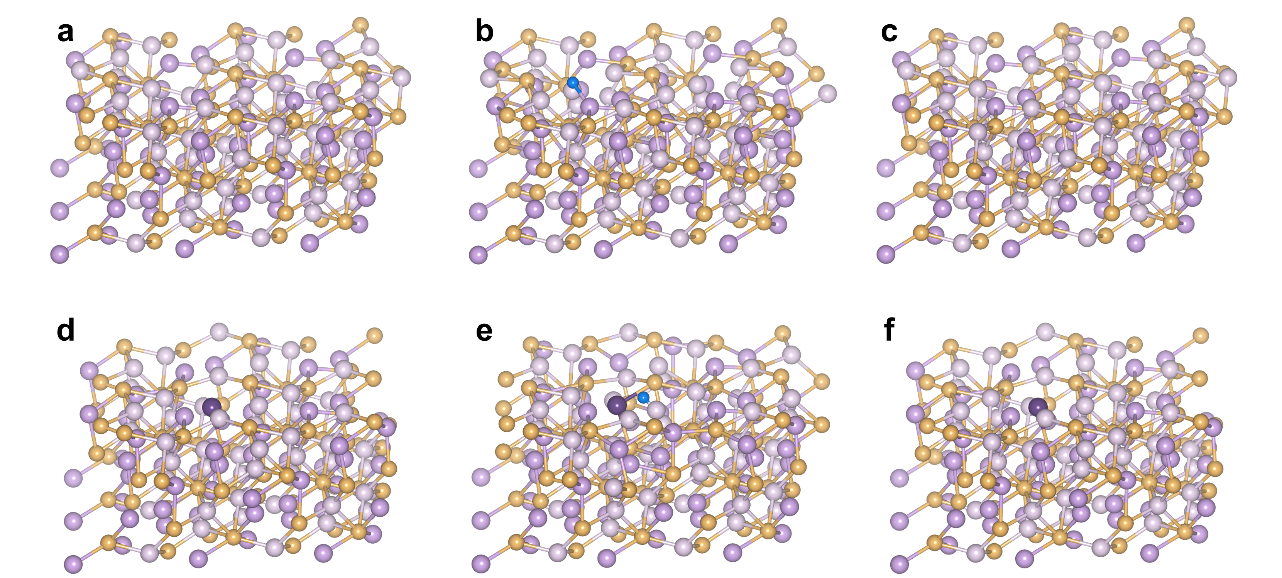


**Supplementary Figure 16.** Theoretical model for DFT calculation of H adsorption and desorption process of the (a-c) NiCoP_v_@NF and (d-f) Cr-NiCoP_v_@NF.


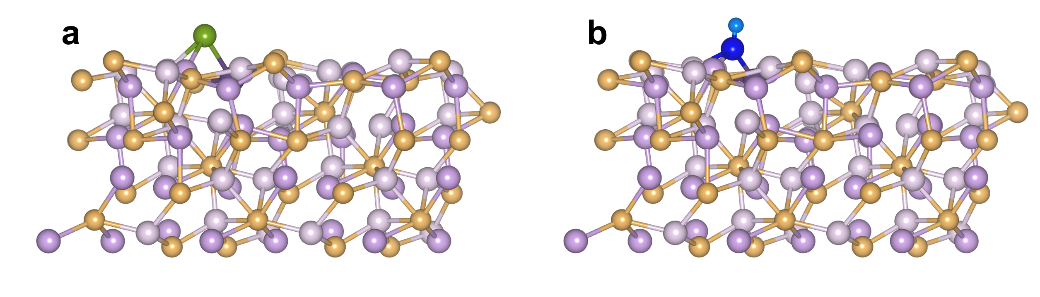


**Supplementary Figure 17.** Theoretical model for DFT calculation of the (a) Cl^-^ and (b) OH^-^ adsorption on the Cr-NiCoP_v_@NF surfaces.


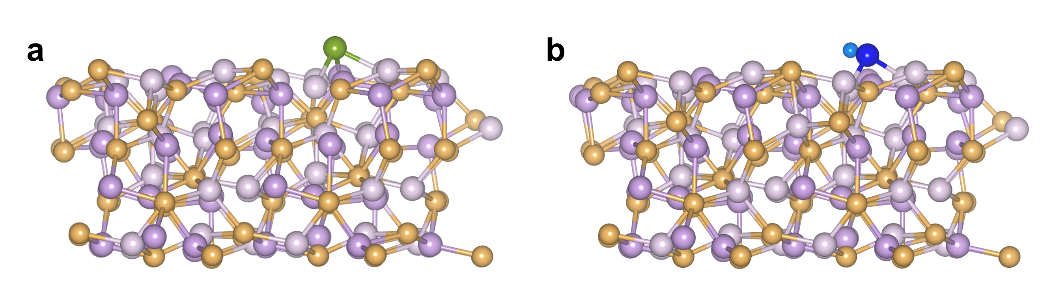


**Supplementary Figure 18.** Theoretical model for DFT calculation of (a) Cl^-^ and (b) OH^-^ adsorption on the NiCoP_v_@NF surfaces.


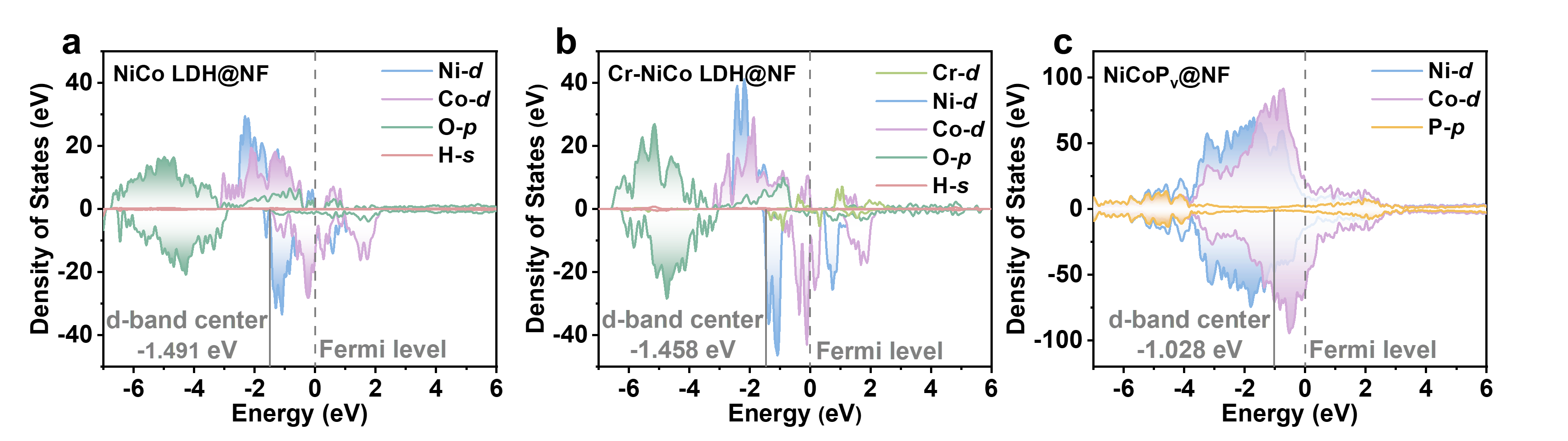


**Supplementary Figure 19.** Density of states of the (a) NiCo LDH@NF, (b) Cr-NiCo LDH@NF, and (c) NiCoP_v_@NF.

**Supplementary Figure 20.** Comparison of Cl^-^ and OH^-^ adsorption energy on NiCoP_v_@NF surfaces. The insets are structure models of the NiCoP_v_@NF.


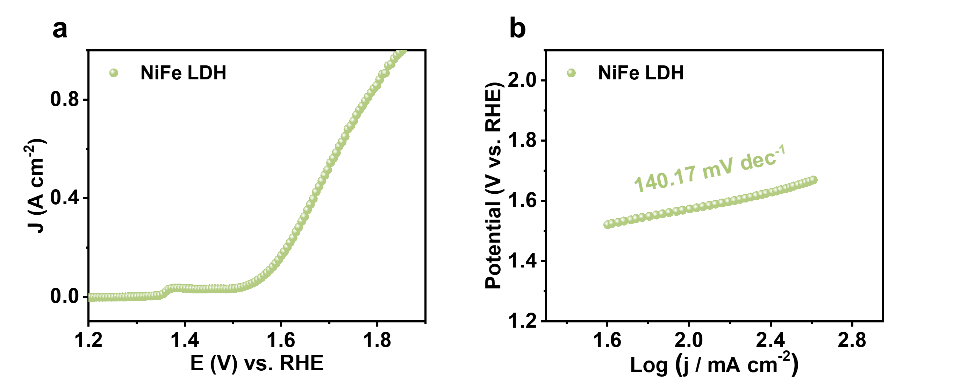


**Supplementary Figure 21.** Electrochemical performance of the NiFe LDH in 1.0 M KOH + seawater electrolyte. (a) Representative OER polarization curves. (b) Tafel slopes.

**Supplementary Figure 22.** Three-electrode measured V-t curve with 10-min start-shutdown cycles at the current density of 0.1 A cm^-2^ at HER period with Cr-NiCoP_v_@NF as the cathode and graphite rod as the anode in 1.0 M KOH + seawater electrolyte.

**Supplementary Figure 23.** Intermittent stability test in the AEM electrolyser (with Cr-NiCoP_v_@NF cathode and NiFe LDH anode) recorded at 0.5 A cm^-2^ in 1.0 M KOH + seawater electrolyte with 12-h start-shutdown cycles at 80 °C.


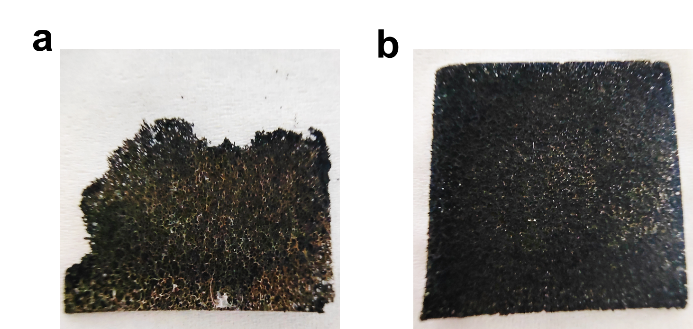


**Supplementary Figure 24.** Photograph of the (a) NiFe LDH anode and (b) Cr-NiCoP_v_@NF cathode after intermittent stability test in the AEM electrolyser at 0.5 A cm^-2^ in 1.0 M KOH + seawater electrolyte with 12-h start-shutdown cycles at 80 °C lasting approximately 120 hours.


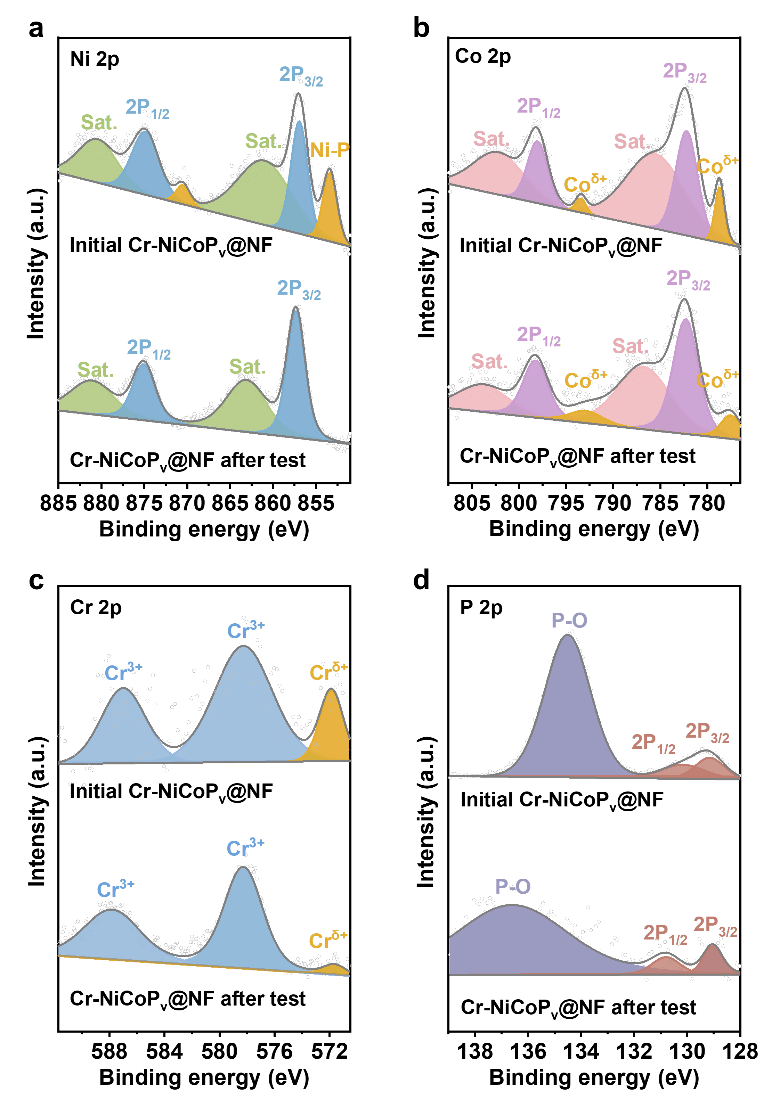


**Supplementary Figure 25.** Comparison of high-resolution (a) Ni 2p, (b) Co 2p, (c) Cr 2p, and (d) P 2p XPS spectra of the Cr-NiCoP_v_@NF before and after the long-time intermittent stability test. The system was in the shutdown period at the end of the intermittent stability test.

**Supplementary Figure 26.** Raman spectra of the Cr-NiCoP_v_@NF before and after the long-time intermittent stability test. The system was in the shutdown period at the end of the intermittent stability test.

**Supplementary Figure 27.** Chronopotentiometry curve using the Cr-NiCoP_v_@NF as anode and graphite rod as cathode operated at 0.1 A cm^-2^ in 1.0 M KOH + seawater electrolyte.

**Supplementary Figure 28.** Quantification of hypochlorites (ClO^−^) generated after 100 cycles (10 min start with 0.5A cm^-2^ +10 min shutdown as 1 cycle) in 1.0 M KOH + seawater electrolyte.

| **Cathode catalyst** | **Electrolyte solution** | **η_10_ (mV)** | **η_100_ (mV)** | **η_1000_ (mV)** | **References** |
| --- | --- | --- | --- | --- | --- |
| Cr-NiCoP_v_@NF | 1.0 M KOH +Seawater | 110 | 178 | 333 | This work |
| NiSe_2_ | 1.0 M KOH +Seawater | 154.5 | - | - | [6] |
| Zr-Ni_2_P/NF | 1.0 M KOH +Seawater | - | 197 | - | [7] |
| WS_2_/WN@CM | 1.0 M KOH +Seawater | 113 | - | - | [8] |
| HfNi-WO_3_@NF | 1.0 M KOH +Seawater | 136 | - | - | [9] |
| CoS_2_@CoFe-LDH | 1.0 M KOH +Seawater | - | 311 | - | [10] |
| Cu@1T-N-W NSs | 1.0 M KOH +Seawater | 158.2 | - | - | [11] |
| CoFeOF/NF | 1.0 M KOH +Seawater | 220 | 280 | 380 | [12] |
| Co_3_(PO_4_)_2_@MXene | 1.0 M KOH +Seawater | 58.6 | 182.9 | - | [13] |
| Co-P-W | 1.0 M KOH +Seawater | 143 | - | - | [14] |
| B-NiCoP | 1.0 M KOH +Seawater | 98 | 190 | - | [15] |
| CoMnTe_2_-1 | 1.0 M KOH +Seawater | 136 | - | - | [16] |
| NiFe-LDH/Cu_2_S | 1.0 M KOH +Seawater | 155.9 | - | - | [17] |
| NiFeS/NF | 1.0 M KOH +Seawater | - | 217 | - | [18] |
| Mo_0.84_Ni_0.16_/MoNiO_x_ | 1.0 M KOH +Seawater | 157.5 | - | - | [19] |

**Supplementary Table 1.** Comparison of HER activity of Cr-NiCoP_v_@NF with other reported HER electrocatalysts in alkaline seawater.

**Supplementary Table 2.** ICP-MS analysis of electrolyte (1.0 M KOH + seawater) after a long time of intermittent electrochemical test.

| **Electrochemical test electrode** | **Electrolyte** | **ICP-MS analysis elements** | **Concentration (ppm)** |
| --- | --- | --- | --- |
| Cathode: Cr-NiCoP_v_@NF (1 cm^2^)  Anode: Graphite rod | 1.0 M KOH + seawater (50ml) | Cr in electrolyte | 0.00026 |

**Supplementary Note 1.** The structural configuration and operational dynamics of the alkaline exchange membrane (AEM) electrolyzer.

The system architecture comprises multiple functional layers: outermost insulating plates ensure electrical isolation and operational safety, followed by current collectors that facilitate uniform current distribution throughout the electrode surface. The porous titanium (Ti) support layer is adjacent to the current collector, which allows for the efficient transport of gases and electrolyte. Gaskets serve as seals to prevent electrolyte leakage and ensure proper gas separation. The cathode and anode catalyst layers are positioned on either side of the AEM. AEM as a separator allows for the selective transport of ions while preventing the mixing of gases. The electrolyte flows into the system through designated inlets on both the cathode and anode sides. At the cathode, the electrolyte undergoes reduction, producing hydrogen gas (H_2_), which exits the system along with the electrolyte through the cathode outlet. Conversely, at the anode, the electrolyte undergoes oxidation, generating oxygen gas (O_2_), which is expelled through the anode outlet along with the electrolyte. This design ensures efficient electrolysis and gas separation.

**Supplementary Note 2.** Calculation of AEM electrolyzer efficiency and H_2_ cost.

The calculations refer to other literature^[20, 21]^.

**Electrolyzer efficiency**

H_2_ production rata @ current density (0.1 A cm^-2^)

= (j A cm^-2^)×(1e^-^/1.602×10^-19^ C)×(1 H_2_/2e^-^)

= 0.1 A cm^-2^/(1.602×10^-19^ C )×(1 H_2_/2)

= 5.18×10^-7^ mol H_2_ cm^-2^ s^-1^

LHV of H_2_

= 120 kJ g^-1^ H_2_

= 2.42×10^5^ J mol^-1^ H_2_

H_2_ power out

= (5.18×10^-7^ mol H_2_ cm^-2^ s^-1^)×(2.42×10^5^ J mol^-1^)

= 0.1254 W cm^-2^

Electrolyzer Power

= (Current density A cm^-2^)×(Corresponding cell voltage V)

= 0.1 A cm^-2^×1.62 V

= 0.162 W cm^-2^

Efficiency of AEMWE

= (H_2_ Power Out)/(Electrolyzer Power)

= 0.1254 W cm^-2^/0.162 W cm^-2^

= 77.4%

**Price of per gasoline-gallon equivalent (GGE) H_2_**

= 1GGE H_2_/H_2_ production rate×Electrolyzer power×Electricity bill

= 0.997 kg/(5.18×10^-7^ mol H_2_ cm^-2^ s^-1^×2kg/mol) ×0.162 W cm^-2^×$ 0.02/kW h

= $0.87 /GGE H_2_ (Compare with $ 2/GGE H_2_ of the U.S. Department of Energy (DOE) by 2026).

**References**

[1] Ding X. *et al.*, Remote Synergy between Heterogeneous Single Atoms and Clusters for Enhanced Oxygen Evolution. *Nano Lett.* **23**, 3309-3316 (2023).

[2] McCrory C. C. L., Jung S., Peters J. C., Jaramillo T. F., Benchmarking Heterogeneous Electrocatalysts for the Oxygen Evolution Reaction. *J. Am. Chem. Soc.* **135**, 16977-16987 (2013).

[3] Kresse G., Joubert D., From ultrasoft pseudopotentials to the projector augmented-wave method. *Phys. Rev. B* **59**, 1758-1775 (1999).

[4] Perdew J. P., Burke K., Ernzerhof M., Generalized Gradient Approximation Made Simple. *Phys. Rev. Lett.* **77**, 3865-3868 (1996).

[5] Grimme S., Antony J., Ehrlich S., Krieg H., A consistent and accurate ab initio parametrization of density functional dispersion correction (DFT-D) for the 94 elements H-Pu. *J. Chem. Phys.* **132**, 154104 (2010).

[6] Zhang S. *et al.*, Hydrothermally synthesized NiSe_2_ nanospheres for efficient bifunctional electrocatalysis in alkaline seawater electrolysis: High performance and stability in HER and OER. *Mater. Res. Bull.* **189**, 113463 (2025).

[7] Tang W., Zhang X., Yang Y., Sun X., Zr-doped porous Ni_2_P nanoarray as a highly efficient electrocatalyst for hydrogen evolution reaction in alkaline seawater. *Nanotechnology* **36**, 205702 (2025).

[8] Zhao J. *et al.*, Modulating electronic by construction WS_2_/WN heterostructure coupled with N-doped carbon to boost alkaline seawater hydrogen production. *Appl. Surf. Sci.* **680**, 161466 (2025).

[9] Itagi M., Chauhan D., Ahn Y., Binder-Free HfNi-Doped WO_3_ Bifunctional Electrocatalysts for Efficient Seawater Electrolysis. *ACS Appl. Energy Mater.* **8**, 6645-6654 (2025).

[10] Afshan G. *et al.*, Green H_2_ Generation from Seawater Deploying a Bifunctional Hetero-Interfaced CoS_2_-CoFe-Layered Double Hydroxide in an Electrolyzer. *Small* **21**, 2406431 (2025).

[11] Prasanna M., Kwak H. B., Oh M. J., Yoo D. J., Architecting a 1T-phase material with metal NPs enriching HER kinetics in alkaline and seawater electrolytes. *Inorg. Chem. Front.* **11**, 5612-5623 (2024).

[12] Patil S. A. *et al.*, Electrostatically robust CoFeOF nanosheet against chloride for green-H_2_ production in alkaline seawater electrolysis. *Chem. Eng. J.* **480**, 146545 (2024).

[13] Chen H. *et al.*, Constructing built-in electric fields in 2D/2D Schottky heterojunctions for efficient alkaline seawater electrolysis. *Inorg. Chem. Front.* **11**, 6909-6918 (2024).

[14] Yuan S., Wu Y., Chen W., Xu Z., Wang Y., In situ construction of W-doped Co-P electrocatalyst by electrodeposition for boosting alkaline water/seawater hydrogen evolution reaction. *Int. J. Hydrog. Energy* **89**, 1430-1439 (2024).

[15] Nguyen C. N. *et al.*, MOF-Templated Synthesis of Three-Dimensional B-Doped NiCoP Hollow Nanorod Arrays for Highly Efficient and Stable Natural Seawater Splitting. *ACS Appl. Energy Mater.* **6**, 10713-10722 (2023).

[16] Karthikeyan S. C. *et al.*, High-efficiency sustainable energy driven alkaline/seawater electrolysis using a novel hetero-structured non-noble bimetal telluride nanorods. *Mater. Today Nano* **24**, 100412 (2023).

[17] Zhang Y. *et al.*, Cu_2_S Nanorods Decorated with NiFe-Layered Double Hydroxide Nanosheets as Bifunctional Electrocatalysts for Hydrogen Evolution in Alkaline Saline Water/Seawater. *ACS Appl. Nano Mater.* **6**, 9816-9824 (2023).

[18] Chen J. *et al.*, High-efficiency overall alkaline seawater splitting: using a nickel–iron sulfide nanosheet array as a bifunctional electrocatalyst. *J. Mater. Chem. A* **11**, 1116-1122 (2023).

[19] Liu J. *et al.*, Rapid synthesis of NiMo-based electrocatalysts at room temperature for efficient oxygen and hydrogen evolution in seawater. *J. Electroanal. Chem.* **935**, 117311 (2023).

[20] Guo L. *et al.*, Phosphorus Defect Mediated Electron Redistribution to Boost Anion Exchange Membrane‐Based Alkaline Seawater Electrolysis. *Adv. Energy Mater.* **14**, 2400975 (2024).

[21] Zhu J. *et al.*, Frustrated Lewis Pair Mediated f‐p‐d Orbital Coupling: Achieving Selective Seawater Oxidation and Breaking ^*^OH and ^*^OOH Scaling Relationship. *Angew. Chem. Int. Ed.* **64**, e202414721 (2024).
